# Clinical-grade iPSC-derived chondrogenic micropellets for treating advanced cartilage defects

**DOI:** 10.1126/sciadv.adw4911

**Published:** 2025-12-17

**Authors:** Yoojun Nam, Narae Park, Jinhyeok Choi, Kijun Lee, Si Hwa Choi, Jang-Woon Kim, SeonJu Choi, Chang Pyo Hong, Jennifer Lee, Joon-Yong Jung, Soon Nam Oh, Yeri Alice Rim, Ji Hyeon Ju

**Affiliations:** ^1^ YiPSCELL Inc., L2 Omnibus Park, 222, Banpo-daero, Seocho-gu, Seoul 06591, Republic of Korea.; ^2^ Department of Biohealth Regulatory Science, Sungkyunkwan University, Suwon, Republic of Korea.; ^3^ CiSTEM Laboratory, Catholic iPSC Research Center, Seoul St. Mary’s Hospital, College of Medicine, The Catholic University of Korea, Seoul 06591, Republic of Korea.; ^4^ Division of Rheumatology, Department of Internal Medicine, Seoul St. Mary’s Hospital, Institute of Medical Science, College of Medicine, The Catholic University of Korea, Seoul 06591, Republic of Korea.; ^5^ Department of Radiology, Seoul St. Mary's Hospital, College of Medicine, The Catholic University of Korea, Seoul, Republic of Korea.

## Abstract

Induced pluripotent stem cell (iPSC)–derived chondrogenic tissues represent a promising alternative for treating cartilage defects in chronic degenerative joint conditions such as osteoarthritis (OA). Cartilage tissue has limited self-repair capacity, and although allogeneic transplantation has potential, a less invasive delivery method could enhance the efficacy of cell-based therapies. The aim of this study was to develop iPSC-derived “minimal injectable unit” chondrogenic micropellets (MIUChons) for delivery via intra-articular injections for OA therapy. To create transplantable allogeneic cartilage tissue, we optimized good manufacturing practice or clinical-grade production of iPSC-derived injectable chondrogenic spheroids and tested them in OA animal models. MIUChons were delivered to damaged cartilage through a single injection. In vivo and in vitro analyses demonstrated that MIUChon treatment effectively reduced cartilage degeneration and deterioration. In addition, injecting MIUChons into the intra-articular cavity improved arthritis symptoms. Overall, MIUChons offer a strategy for treating cartilage deterioration via intra-articular injection in patients with OA.

## Teaser

Injectable iPSC-derived chondrogenic micropellets advance cartilage repair and osteoarthritis treatment.

## INTRODUCTION

Stem cell–based therapeutics provide a previously unknown approach for repairing treating and regenerating damaged tissues (*1*, *2*). Cartilage is of particular interest in regeneration due to its relatively simple structure, consisting of an extracellular matrix (ECM) and chondrocytes (*3*, *4*). Cartilage damage typically results from injury or gradual degeneration and often culminates in osteoarthritis (OA) (*5*). While OA was historically regarded as mere mechanical “wear and tear” of load-bearing joints, it is now recognized as a multifactorial disease in which various cells such as chondrocytes, synovial cells, and the subchondral bone are actively involved. Moreover, large-scale genome-wide association studies indicate that OA has a substantial heritable component (estimated narrow-sense heritability 40 to 60%), with risk alleles identified in genes encoding cartilage-specific ECM regulators (e.g., *GDF5* and *COL11A1*) and key members of the transforming growth factor–β (TGF-β)/SMAD3 signaling pathway, all of which predispose individuals to accelerated cartilage degeneration and OA progression (*6*–*8*). The disrupted chondrocyte mechanosignaling actively drives cartilage degradation through inflammatory and metabolic pathways, leading to reduced joint mobility and, in severe cases, the need for artificial joint replacement. The rising prevalence of OA in an aging population underscores the urgent need for effective treatments (*9*). Although transplantation of in vitro–expanded chondrocytes or adult stem cells has been explored, these techniques highlight the need for alternative approaches, such as pluripotent stem cell–derived cartilaginous tissue or cell-based therapies, to achieve more consistent and durable cartilage protection or regeneration (*1*, *10*).

Induced pluripotent stem cell (iPSC)–based chondrogenesis has been developed and analyzed in preliminary studies, suggesting its potential as a therapeutic agent for treating damaged cartilage (*11*, *12*). Human iPSCs, originally developed by Takahashi and Yamanaka in 2006, have transformed regenerative medicine by offering donor-specific stem cells with the versatility to generate numerous specialized lineages, such as chondrocytes (*13*). Over the past decade, research has increasingly focused on applying iPSCs for cartilage regeneration, given their potential to overcome limitations in current treatments, such as low cell yield and chondrocyte dedifferentiation (*14*–*18*). The ability to generate chondrocytes from iPSCs has opened avenues for cartilage regeneration, offering an unlimited supply of autologous or allogeneic cells for therapy (*19*).

Research indicates that iPSC-derived chondrocytes are effective in facilitating cartilage repair. In osteochondral defects of immunosuppressed rats, these cells yielded notably improved restoration compared to control groups (*20*). Meanwhile, uniform cartilaginous constructs produced from chondrocyte-specific reporter hiPSC lines, when transplanted into joint surface defects in both immunodeficient rats and immunosuppressed minipigs, successfully survived, integrated into surrounding cartilage, and displayed no tumor development (*21*).

As the field progresses, research has shifted toward three-dimensional (3D) culture systems and chondrogenic spheroids, which more accurately mimic the native cartilage environment (*14*). Chondrogenic spheroids derived from iPSCs maintained a cartilage-like phenotype in vivo, closely resembling chondrogenic-like tissues generated from the same spheroids in vitro (*22*). In contrast, spheroids derived from bone marrow mesenchymal stem cells (BM-MSCs) formed distinct bone-like tissues. This suggests that iPSC-derived chondrogenic spheroids may form cartilage-like tissues without undergoing endochondral ossification, making them promising candidates for treating cartilage defects in vivo. In addition, their rapid fusion capability enables chondrogenic spheroids to serve as building blocks for constructing larger cartilage tissues using methods such as bioprinting (*23*). However, surgical delivery poses challenges, and a less invasive method could enhance the effectiveness of current therapeutic approaches.

In this study, we established a reproducible, clinical-grade protocol for generating minimal injectable unit chondrogenic micropellets (MIUChons) derived from iPSCs, aiming to treat cartilage defects via intra-articular (IA) injections. We optimized the differentiation process to produce MIUChons with high expression of essential chondrogenic markers, such as type II collagen and SOX9, which are critical for cartilage development. Genetic stability analyses confirmed the absence of notable somatic mutations or copy number variations. In addition, implanting MIUChons into immunodeficient mice demonstrated a lack of tumorigenicity. Single-cell RNA sequencing (scRNA-seq) mapped the differentiation process, highlighting robust chondrocyte development and ECM-related functions.

The therapeutic potential was tested in various animal models, including rats, rabbits, beagles, and minipigs. Results showed that MIUChon injections alleviated cartilage degradation and promoted cartilage regeneration. Overall, our findings suggest that MIUChons can effectively reduce OA symptoms and improve cartilage quality, making them a promising candidate for clinical applications in cartilage regeneration therapies. As we advance toward human trials, iPSC-derived MIUChons could revolutionize the treatment of OA and other cartilage-related disorders.

## RESULTS

### Development of a clinical-grade protocol to efficiently generate reproducible MIUChons

**Fig. 1.**
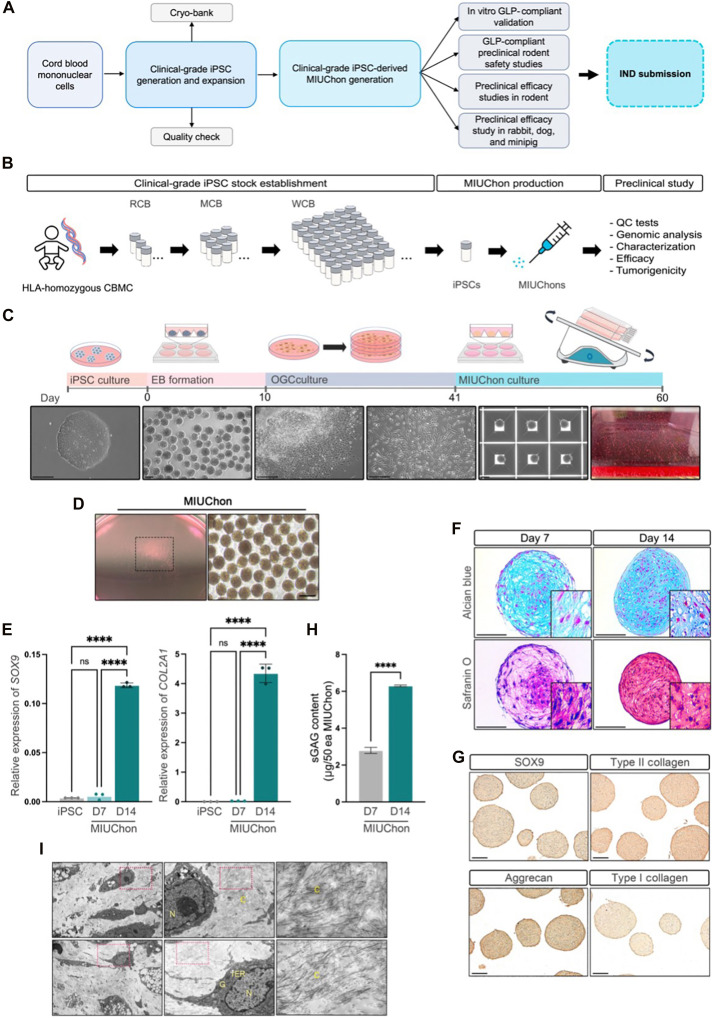
The end-to-end manufacturing scheme and characterization of clinical-grade MIUChons. (**A**) Workflow illustrating a pipeline to manufacture and test clinical-grade MIUChons to submit a phase 1 clinical trial investigational new drug application with the Korean Food and Drug Administration. (**B**) An overview of the preclinical study of MIUChon. (**C**) The differentiation process for MIUChon generation. Scale bars, 200 μm. (**D**) Morphology of MIUChons after differentiation. Scale bars, 200 μm. (**E**) Increased *SOX9* and type II collagen (*COL2A1*) expression in day 7 (D7) and final day 14 (D14) MIUChons compared to iPSCs. (**F**) Increased intensity of alcian blue and safranin O in D7 and final D14 MIUChons. Scale bars, 100 μm. (**G**) SOX9, type II collagen, aggrecan, and type I collagen expression in final D14 MIUChons. Scale bars, 100 μm. (**H**) Increased soluble GAG content at day 14 of differentiation compared to D7 MIUChons. (**I**) Transmission electron microscopy images of MIUChons. N, nucleus; ER, endoplasmic reticulum; G, Golgi complex; C, collagen fiber. Significance: ****P < 0.00011; ns, not significant.

We established an integrated pipeline for the end-to-end development of clinical-grade MIUChons, encompassing iPSC generation, cell bank establishment, in vitro functional validation, and in vivo testing (Fig. 1, A and B). MIUChons were differentiated through four steps: 1) iPSC culture, 2) embryoid body formation, 3) outgrowth cell (OGC) induction, and 4) Chondrogenic differentiation (MIUChon culture) (Fig. 1C). The established protocol enabled efficient MIUChon generation (Fig. 1D). Hyaline cartilage primarily consists of type II collagen, and the transcription factor SOX9 is a key regulator of its secretion during chondrogenic differentiation (*24*, *25*). *SOX9* and type II collagen (*COL2A1*) gene expression increased significantly in the final day 14 MIUChons compared to the original iPSCs or day 7 differentiated cells (Fig. 1E). The generated MIUChons exhibited a time-dependent increase in ECM production during in vitro chondrogenesis (Fig. 1F). Positive expression of *SOX9* and *COL2A1* was confirmed in the chondrogenic micropellets (Fig. 1G). In addition, glycosaminoglycan (GAG) secretion was significantly higher in the final products compared to day 7 differentiated cells (Fig. 1H). As chondrocytes secrete abundant ECM proteins, the endoplasmic reticulum (ER)–Golgi and fibrous structures were prominently observed in MIUChons (Fig. 1I).

To further characterize the generated MIUChons, we compared their chondrocyte phenotype to that of native articular chondrocytes. An integrated scRNA-seq analysis using publicly available datasets from healthy human knee cartilage [GSE220243 (*26*) and GSE255460 (*27*)] alongside our MIUChon dataset (CL2) was performed to evaluate the transcriptomic similarity between chondrocytes derived form MIUChon and native articular cartilage (fig. S1A). Datasets were SCTransform-normalized and integrated via Seurat’s IntegrateLayers using canonical correlation analysis (CCA) to correct batch effects. Chondrocyte subtypes were classified based on previously defined marker genes (*26*, *28*). Uniform manifold approximation and projection (UMAP) analysis of the integrated dataset identified 10 transcriptionally distinct chondrocyte subtypes, including reparative chondrocytes (RepC), fibrocartilage chondrocytes (FC1/FC2), regulatory chondrocytes (RegC), effector chondrocytes (EC), proliferative chondrocytes (ProC), and hypertrophic chondrocytes (HTC) (fig. S1, B and C). Comparative analysis of subtype proportions revealed that MIUChon-derived cchondrocytes overlapped with subtypes previously defined in native cartilage (fig. S1, D and E). Notably, MIUChon showed a higher proportion of RepC and FC1 subtypes compared to native cartilage, while the native samples exhibited relatively greater proportions of RegC, preHTC, ProC, HomC, and HTC subtypes. Given these compositional differences, we further examined the expression of key chondrogenic markers. *SOX9* was broadly expressed across MIUChon, whereas *COL2A1* expression was predominantly enriched in the RepC subtype. Such an expression pattern highlights the activation of chondrogenic programs in MIUChon, particularly in reparative chondrocyte populations. Together, these findings demonstrate that, despite compositional differences, MIUChon-derived single cells share key transcriptomic features with reparative and ECM-producing subpopulations found in native cartilage, supporting their molecular resemblance to functional chondrocytes and providing a rationale for their therapeutic potential in cartilage regeneration.

Tumorigenicity is a critical concern for stem cell–based therapeutics. The absence of residual iPSCs in MIUChons was confirmed by the negative expression of TRA-1-60, a representative marker of iPSCs (fig. S2A). To further confirm the absence of tumorigenicity, iPSCs, progenitor outgrowth cells (OGCs), or MIUChons were implanted into immunodeficient mice (fig. S2B). Histological analysis showed no evidence of teratoma formation in OGCs and MIUChons (fig. S1C). The iPSCs derived from the master cell bank (MCB) passed rigorous qualifying tests (table S1), and the final generated MIUChons were characterized according to their specific release criteria (table S2).

The genetic stability of MIUChons was assessed by analyzing short tandem repeats (STRs), somatic mutations, and copy number variations (CNVs). STR analysis demonstrated genetic homogeneity among iPSCs from the MCB, working cell bank (WCB), and MIUChons at STR loci (fig. S3). Whole-genome sequencing data revealed somatic coding variants with potential functional effects in MIUChons, averaging 3291 variants across three replicates compared to the WCB. However, the frequency of these variants was low (fig. S4A). Among these, 13 variants (0.56%) were detected between the MCB and WCB, whereas 6 to 10 variants (0.25 to 0.27%) were found between the WCB and MIUChons. Two somatic coding variants across the MCB, WCB, and MIUChons were predicted to impair protein structure using PolyPhen2 and sorting intolerant from tolerant (SIFT) (fig. S4B). No CNVs were observed in the MCB, WCB, or MIUChons (fig. S5A), and 20q11.21, a recurrent genomic aberration in human pluripotent stem cells, was absent (fig. S5B) (*29*, *30*). These results demonstrate that the manufactured MIUChons maintain the required genetic stability for therapeutic use.

### Characterization of MIUChons as functional chondrospheroids exhibiting chondrocyte maturation and ECM-related functions

**Fig. 2.**
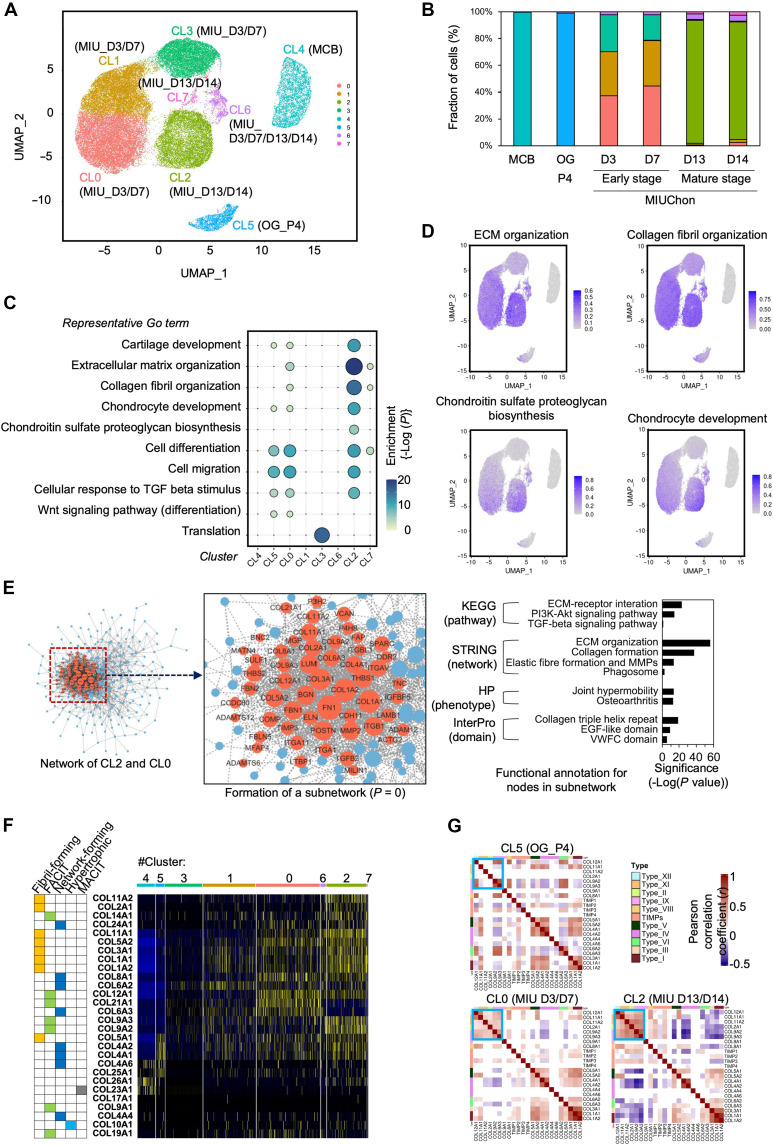
Single-cell transcriptomic analysis of MIUChon during differentiation. (**A**) Uniform manifold approximation projection (UMAP) clustering of iPSC (MCB), OG (fourth passage), and MIUChons differentiated at four time points (Day 3, 7, 13, and 14). (**B**) Bar plot showing the fraction of cells assigned to each cell type during differentiation. (**C**) Gene Ontology (GO) enrichment analysis for highly expressed genes in each cluster. Genes highly expressed in each cluster were annotated using DAVID, and notable GO terms related to cellular process functions were selected with a stringent cutoff of *P* < 0.01. (**D**) Feature heatmap of functional terms related to cartilage ECM organization, collagen fibril organization, chondroitin sulfate proteoglycan biosynthesis, and chondrocyte development. (**E**) Interaction of genes highly expressed in clusters 0 and 2 in MIUChon as determined using the STRING database search. A notable subnetwork (red nodes) was predicted using CytoCluster (*P* = 0). Functional annotation for nodes in the subnetwork was performed using STRING and DAVID with a stringent cutoff of *P* < 0.001. (**F**) Expression heatmap of the collagen gene family at each stage of differentiation. (**G**) Expression correlation of collagens, TIMPs, and MMPs in clusters 5, 0, and 2.

To elucidate the differentiation process from iPSCs into MIUChons and verify their chondrospheroid characteristics, scRNA-seq was performed. The captured cells from each sample are listed in table S3. UMAP clustering analysis revealed cellular heterogeneity with distinct clusters among the MCB, mesenchymal-like OGCs at the fourth passage (OGC_P4), and MIUChons (Fig. 2, A and B). Marker gene analysis indicated pronounced expression of genes related to chondrocyte and cartilage development in both early-stage (clusters 0, 1, and 3) and mature-stage (cluster 2) MIUChons (fig. S6). By performing Gene Ontology (GO) analysis on the differentially expressed genes (DEGs) within each cluster, we identified the enrichment of terms such as “ECM organization” (*P* = 1.7 × 10^−29^), “collagen fibril organization” (*P* = 3.6 × 10^−16^), “chondrocyte development” (*P* = 2.7 × 10^−11^), “chondroitin sulfate proteoglycan” (*P* = 1.7 × 10^−7^), “TGF-β signaling” (*P* = 1.5 × 10^−9^), and “cartilage development” (*P* = 5.3 × 10^−12^) (Fig. 2C). Genes associated with ECM organization showed pronounced expression in clusters 0 and 2 (Fig. 2D). To investigate the transcriptional coordination underlying MIUChon differentiation, we performed network analysis of DEGs in cluster 0 (early-stage MIUChon) and cluster 2 (mature-stage MIUChon). This analysis identified a highly significant subnetwork (*P* = 0) with dense connectivity among genes involved in ECM organization, collagen biosynthesis, and chondrocyte development (Fig. 2E). Notably, the subnetwork linking CL0 and CL2 represents a transcriptional continuum of ECM-related genes spanning early to mature MIUChon stages. This continuum suggests that ECM-related gene programs are not abruptly switched on, but are progressively established and maintained across differentiation stages. The strong interconnectivity of core ECM regulators, including *COL2A1*, *COL11A1*, *COMP*, and *FN1*, further supports the idea that clusters 0 and 2 are functionally linked through shared regulatory networks governing cartilage matrix assembly. Diverse types of collagen genes were predominantly expressed in clusters 0 and 2 (Fig. 2F). This transcriptional activation of diverse collagen genes is essential for cartilage ECM assembly, thereby promoting the structural complexity and functional integrity of the developing matrix. Specifically, fibril-forming collagens (*COL2A1*, *COL11A1/2*, *COL5A1/2*, *COL3A1*, and *COL1A1/2*), fibril-associated collagens with interrupted triple helices (FACIT) (*COL12A1*, *COL14A1*, *COL21A1*, and *COL9A2/3*), and network-forming collagens (*COL6A2/3*, *COL8A1*, *COL24A1*, and *COL4A1/2*) were strongly expressed. In contrast, collagen genes associated with hypertrophic or ossification phases, such as *COL10A1* and *COL23A1*, were expressed at very low levels and in a limited number of cells, suggesting that MIUChon maintains a nonhypertrophic, cartilage ECM-productive chondrocyte phenotype. Furthermore, correlation analysis of collagen gene expression revealed strong positive co-expression patterns among *COL2A1*, *COL12A1*, *COL11A1/2*, and *COL9A2/3* in clusters 0 and 2 (Fig. 2G). This finding supports the presence of coordinated regulatory programs that promote cartilage-specific ECM synthesis. Together, these findings suggest that MIUChon activates a defined ECM transcriptional network characteristic of functional chondrospheroids and sustains the expression of key collagen components throughout differentiation.

### Reparative subpopulations and ECM-centric signaling in fully differentiated final MIUChon

To further resolve the heterogeneity within the mature MIUChon cluster CL2, we performed high-resolution subclustering, identifying six distinct subpopulations (CL2-0 to CL2-5) (fig. S7A). These subclusters were consistently observed in MIUChon samples (D13 and D14), indicating stable composition across replicates (fig. S7B and Fig. 1B). Marker gene expression suggested that CL2-0 and CL2-1 represent reparative chondrocytes (RepC), CL2-2 corresponds to fibrocartilage-like chondrocytes (FC), and CL2-1/2 also co-express regulatory chondrocyte (RegC) markers (fig. S7C). GO enrichment analysis showed that CL2-0 and CL2-1 were enriched in pathways related to cartilage development and ECM organization, with CL2-0 also selectively enriched in cell adhesion, migration, and signal transduction (fig. S7D). These clusters exhibited high expression of cartilage-associated genes, including *COL2A1*, *ACAN*, *MATN4*, *SOX9*, *COMP*, and *PTN* (fig. S7E).

To investigate intercellular communication within CL2, we applied CellChat analysis, which revealed that CL2-0 and CL2-1 act as major signaling hubs. These clusters showed extensive ligand-receptor interactions with other subpopulations via ECM-related ligands such as FN1, COLLAGEN, PERIOSTIN, and PTN (fig. S7, F and G). Notably, CL2-0 displayed the strongest outgoing signaling, while CL2-1 exhibited both strong outgoing and incoming activity, suggesting complementary roles in signal transmission. Network analysis further confirmed that these ligand-receptor modules are enriched in key cartilage repair pathways, including ECM assembly, collagen trimerization, and integrin-mediated cell-matrix signaling (fig. S7, H and I). Collectively, these findings suggest that CL2 comprises functionally distinct subpopulations, with CL2-0 and CL2-1 serving as reparative cores and other clusters contributing to regulatory and structural support through coordinated intercellular signaling.

### MIUChon injections relieve cartilage destruction and exhibit possible direct regeneration in OA in vivo

**Fig. 3.**
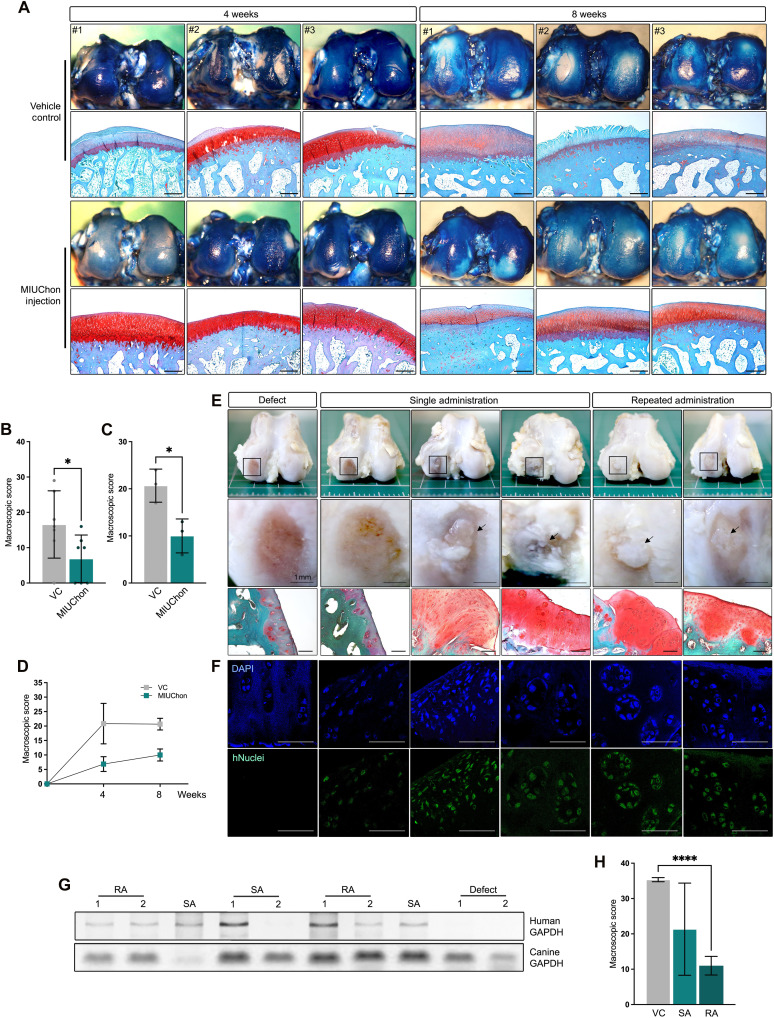
In vivo chondroprotective ability evaluation in rabbit and canine OA models. (A) to (D) present the experimental results from the rabbit model, while (E) to (H) show the results from the beagle dog model. More detailed information can be found in fig. S8. (**A**) Staining images of ACLT-induced articular cartilage 4 and 8 weeks after MIUChon injection. (**B**) Macroscopic score of the 4-week group. (**C**) Macroscopic score of the 8-week group. (**D**) Combined graph showing the macroscopic score in both groups. (**E**) Histological image of canine ACLT-induced articular cartilage injected with MIUChon. (**F**) An image of stained human nuclei in the canine articular cartilage. Positive expression is confirmed in the MIUChon-injected groups. (**G**) A gel image of human and canine *GAPDH* expressed in isolated canine cartilage tissue. RA, repeated administration; SA, single administration. (**H**) The macroscopic score of canine OA models injected with MIUChons. Scale bars, 200 μm. (A) to (D) present the experimental results from the rabbit model, while (E) to (H) show the results from the beagle dog model. More detailed information can be found in fig. S8.

The in vivo efficacy of MIUChon was first established in a rat OA model. OA was induced by IA injection of monosodium iodoacetate (MIA) into 13-week-old rats, and MIUChon was administered 4 weeks later (fig. S8A). Joint tissues were harvested for analysis 4 weeks after treatment. In the MIA-induced OA model, cartilage that received MIUChon displayed markedly stronger safranin O and toluidine blue staining compared to controls (fig. S9A). Despite the improved histochemical staining, immunoreactivity for aggrecan and type II collagen remained comparable to that in controls. This is likely because 1) MIA depletes aggrecan within 1 to 2 weeks, and 2) the rat knee joint contains < 0.1 ml of synovial fluid, severely restricting both the therapeutic time window and surface contact area available to the 150- to 200-μm MIUChon. Notably, the articular cartilage layer was thicker in the MIUChon-treated group (fig. S9B). To confirm whether human iPSC-derived MIUChon had engrafted into rat cartilage, we probed for human nuclei. No signal was detected in control cartilage, whereas multiple cells and cluster-like structures in the knees of MIUChon-treated rats stained positive for human nuclei (fig. S9C). To overcome the biomechanical limitations inherent to the rat model, we next assessed MIUChon efficacy in large-animal anterior cruciate ligament transection (ACLT) models (rabbits, beagle dogs, and minipigs) that exhibit slower disease progression and have a substantially larger IA volume (see Fig. 3).

The therapeutic effects of MIUChons were further validated in a rabbit model of ACLT-induced OA. ACLT was performed on 8-month-old rabbits, and OA was induced 4 weeks later, before MIUChon injection. Articular cartilage recovery was evaluated at 4 and 8 weeks postinjection (fig. S8B). MIUChon-injected rabbits exhibited improved cartilage morphology in the femoral cartilage 4 weeks postinjection (Fig. 3A). By 8 weeks, the vehicle control group showed notable cartilage degradation, whereas the MIUChon-injected group displayed improved cartilage condition. Representative images of femoral and tibial cartilages are shown in fig. S10. Microscopic scores at both time points indicated remarkable improvement in OA at 4 (Fig. 3B) and 8 weeks (Fig. 3, C and D) postinjection.

Direct cartilage recovery was also observed in rabbit osteochondral defects. Despite the severe cartilage damage caused by the induced defects, implanted MIUChons exhibited chondrogenic activity in vivo. The integrated MIUChons displayed lacunae-like morphologies in the cartilage and intense safranin O staining (fig. S11).

Although the initial findings were promising, the small joint cavity and low dosage of injected MIUChons were constraints that hindered a comprehensive evaluation of their regenerative potential. Furthermore, a more advanced OA model was needed to better replicate advanced OA-like (KL grade III-IV–equivalent) conditions, where MIUChon efficacy could be further demonstrated. To address this, the therapeutic effects of MIUChon were evaluated in beagle OA models using a combination of ACLT and medial meniscectomy (MMx) to induce severe cartilage defects (fig. S8C). We first induced ACLT in the beagle knee joint and partially removed one part of the corresponding meniscus (MMx) to induce a more severe cartilage defect (fig. S12A). The meniscus removed from each animal is shown in fig. S12B. Four weeks post-surgery, MIUChons were injected into the knee joints of 15-month-old beagle dogs. Repeated MIUChon administration was tested in two dogs.

ACLT and MMx successfully induced severe cartilage damage, and in control knees, subchondral bone exposure was observed 14 weeks postinjection (Fig. 3E). Of the three dogs receiving a single MIUChon injection, two showed improved OA. Both dogs receiving two injections demonstrated cartilage recovery, with histological analysis revealing MIUChon-like cluster morphologies and intense safranin O staining in the defective areas. Positive staining for human nuclei confirmed the integration of MIUChons into canine cartilage (Fig. 3F). Gene expression analysis near the defective area showed human *GAPDH* expression in canine cartilage tissues injected with MIUChons, whereas the ACLT with MMx control lacked this signal (Fig. 3G). The presence of human-specific molecules after MIUChon treatment in ACLT animal models was also supported by both rabbit proteome data and minipig transcriptome data (fig. S13, A and B). GO enrichment analysis of human-specific proteins (502) identified in rabbits and human-specific transcripts (208) identified in minipigs revealed significant enrichment of regenerative functional categories such as cytoskeleton organization, ECM remodeling, and tissue regeneration (fig. S13C). These findings suggest that human-derived sequences persisted after MIUChon treatment and actively contributed to the regenerative process of damaged cartilage tissues.

Although one subject had a macroscopic score comparable to the defect control group, repeated MIUChon administration resulted in notable improvements in the cartilage condition. Macroscopic scores showed no significant differences overall (Fig. 3H); however, significant differences were observed between the defect and repeated-administration groups. These results demonstrated the efficacy of MIUChons in improving OA in various animal models, including larger animals, suggesting their potential for direct cartilage regeneration.

To further explore the mechanism of action (MoA) of MIUChon in vivo, we performed a proteomic profiling using knee cartilage tissues collected from MIUChon-treated (M) and untreated ACLT (A) rabbits (fig. S14, A to C). GO enrichment analysis of proteins that were up-regulated (≥2-fold) in the MIUChon-treated group revealed enrichment in functional categories associated with 1) cell engraftment and cytoskeletal reorganization, 2) protein synthesis and organelle stress response regulation, 3) ECM restoration and chondrogenic gene expression, 4) intercellular signaling and growth factor modulation, 5) enhanced cell survival, and 6) functional recovery and long-term tissue remodeling (fig. S16, D and E, and table S6). These findings suggest that MIUChon not only engrafts into the damaged cartilage, but also contributes to ECM remodeling, activation of chondrogenic programs, and stabilization of tissue homeostasis. Consistently, protein profiling of growth factors and cytokines (fig. S16, F and G) revealed increased levels of regenerative factors (e.g., TGFβ3 and BMP2), elevated anti-inflammatory cytokines including interleukin-4 (IL-4), IL-13, and members of the TGF-β family, and decreased expression of proinflammatory cytokines such as IL-6, tumor necrosis factor–α (TNF-α), IL-17A, and CXCL10. These results support a paracrine mechanism that promotes a proregenerative immune environment. Collectively, these findings provide complementary in vivo evidence supporting the therapeutic role of MIUChon through both direct engraftment and paracrine signaling.

### MIUChon promotes cartilage regeneration in a minipig OA model

Rat, rabbit, and canine OA models confirmed the cartilage-protective effects of MIUChons. The direct attachment of MIUChons to cartilage suggested possible regeneration in the beagle model. To evaluate the effects of MIUChons in a larger animal model using the full human clinical dose, we optimized ACLT and partial MMx to induce severe OA in minipigs, similar to the beagle models (fig. S8D). Body weight measurements were recorded at three time points: before injection, and at 8 and 24 weeks postinjection. No significant changes or differences in body weight were observed between the experimental groups (fig. S15A).

Behavioral evaluations were attempted in the minipig models to assess arthritic pain, one of the primary symptoms of OA. Dynamic weight bearing (WB), which measures the weight difference exerted by each foot on a sensor floor, was used to evaluate nociception at several time points (fig. S15B). Similar to body weight, no significant differences in weight distribution of the defective knee were observed between the groups.

**Fig. 4.**
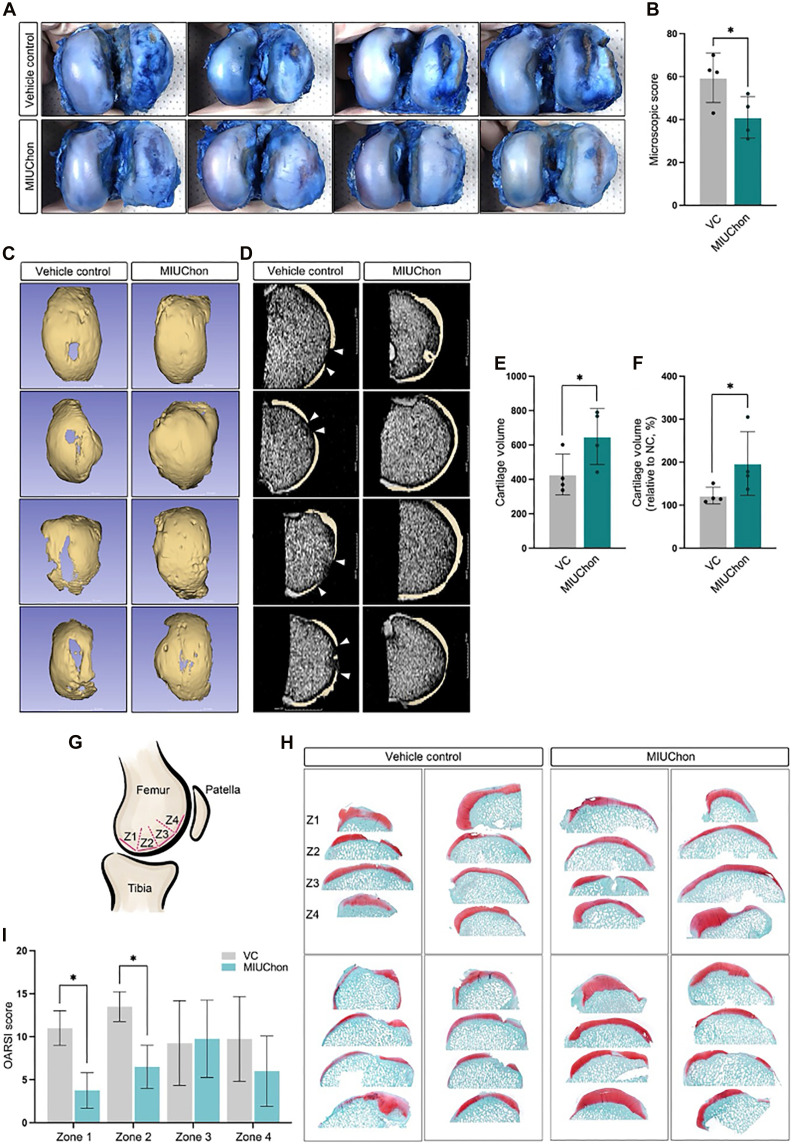
In vivo chondroprotective ability evaluation in the minipig OA model. (**A**) Evans blue–stained femoral articular cartilage of minipig knee joints. (**B**) Macroscopic scoring of femoral articular cartilage in minipig knee joints. (**C**) Three-dimensional reconstructed MRI image of the femoral articular cartilage in minipig knee joints. (**D**) Micro-CT images of femoral articular cartilage in minipig knee joints. (**E**) Total cartilage volume and (**F**) relative cartilage volume ratio evaluation. (**G**) Scheme of analyzed regions (zones 1 to 4) in the minipig articular cartilage. (**H**) A cross-sectional image of the minipig articular cartilage displaying each zone. (**I**) OARSI scoring of each zone in the articular cartilage tissue.

At 16 weeks postinjection, the MIUChon-injected group exhibited less destruction of the articular cartilage compared to the vehicle control (Fig. 4A). Macroscopic scores indicated possible cartilage regeneration in the MIUChon-treated group (Fig. 4B and fig. S16). Joint tissue was assessed through magnetic resonance imaging (MRI) to quantitatively measure and visualize cartilage regeneration (Fig. 4C). 3D imaging of the cartilage surface revealed defects as blank areas in the control group, whereas the majority of the cartilage area in the MIUChon-injected group showed no vacant regions. Sectioned x-ray images further confirmed that defective cartilage in the vehicle group was repaired and filled following a single MIUChon injection (Fig. 4D). MRI analysis demonstrated an increase in total cartilage volume in the MIUChon-treated group compared to controls (Fig. 4E). Reduced cartilage degeneration was evident in the MIUChon-injected group, as confirmed by the relative cartilage volume ratio (Fig. 4F).

In the minipig OA models, specific regions of the articular cartilage exhibited greater efficacy. Four zones (zones 1 to 4) were analyzed (Fig. 4G). The dorsal part of the cartilage (zones 1 and 2) showed significantly better recovery or protection following MIUChon injections (Fig. 4, H and I). These results confirmed the efficacy of MIUChon treatment in a large animal model compared to the vehicle control.

To investigate transcriptomic changes in signaling pathways related to cartilage regeneration, RNA-seq was performed on paraffin block samples from OA-induced (*n* = 4) and MIUChon-treated (*n* = 4) groups. Differential expression (DE) analysis revealed significant enrichment of down-regulated genes associated with ER overload response (*P* = 2.3 × 10^−3^) and programmed cell death (*P* = 6.0 × 10^−4^) in the MIUChon-treated group (fig. S17A). Up-regulated genes were significantly enriched in functional categories related to the WNT signaling pathway (*P* = 9.3 × 10^−4^) and the regulation of the TGF-β receptor signaling pathway (*P* = 3.6 × 10^−3^) (fig. S17B).

Gene set enrichment analysis (GSEA) further validated these findings, showing the enrichment of down-regulated genes associated with ECM disassembly in the MIUChon-treated group (fig. S17, C and D). In addition, genes related to WNT signaling, TGF-β receptor signaling, target of rapamycin (TOR) signaling, and programmed cell death were closely interactive with the MIUChon-associated subnetwork involved in cartilage development (fig. S17E).

### Evaluation of MIUChon efficacy through behavioral assessment of minipigs

**Fig. 5.**
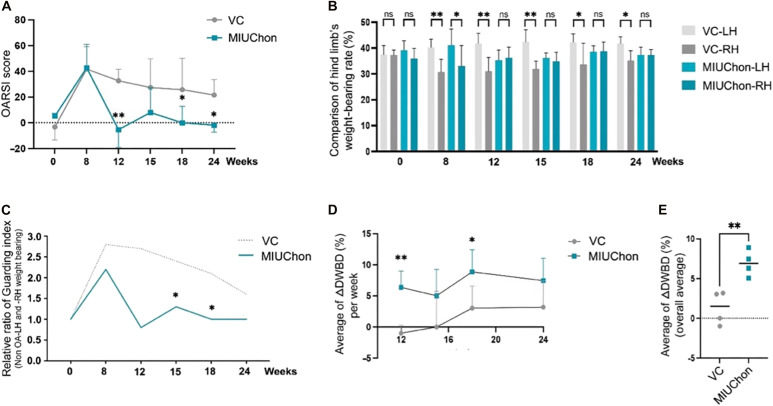
Behavioral assessment of the efficacy in OA minipigs. (**A**) Symmetry index (SI) in the vehicle control group and MIUChon treatment group over time. Increased SI at 8 weeks confirms OA induction, with significant improvement in MIUChon treatment groups at 12, 18, and 24 weeks (one-way ANOVA, **P < 0.01, *P < 0.1). (**B**) Contralateral weight-bearing distribution (CWBD) of the left hindlimb (LH). Significant weight redistribution observed at 8 weeks in both groups. The vehicle control group shows increased LH weight bearing at 12, 15, and 24 weeks. G2 shows no significant difference in weight bearing between limbs posttreatment. (**C**) Guarding index (GI) before and after OA induction. The weight-bearing difference was set to 1 for comparison. (**D**) Individual diagonal weight-bearing distribution (DWBD) changes (ΔDWBD) in the vehicle control group and MIUChon treatment group. Significant changes in G2 at 12 and 18 weeks. (**E**) Comprehensive comparison of ΔDWBD in the vehicle control group and MIUChon treatment group over time.

To further confirm the asymmetry caused by increased weight bearing (WB) in the left hindlimb (LH) due to compensatory weight distribution for the right hindlimb (RH) with induced OA, the symmetry index (SI) was calculated. At 8 weeks post-OA induction, the SI of both the vehicle control group and the MIUChon treatment group similarly increased, indicating greater asymmetry and confirming successful OA induction. In the MIUChon treatment group, the asymmetry significantly improved at 12, 18, and 24 weeks compared to 8 weeks (Fig. 5A). The SI (%) of the vehicle control group and the MIUChon treatment group was analyzed using one-way analysis of variance (ANOVA; **P < 0.01, *P < 0.1).

To assess the redistribution of compensatory WB to the contralateral limb (LH) in response to OA induced in the RH, the contralateral weight-bearing distribution (CWBD) was calculated. At 8 weeks post-OA induction, both the vehicle control group and the MIUChon treatment group showed significant WB redistribution, further confirming successful OA induction. Subsequently, the vehicle control group showed a near-significant *P* value (*P* = 0.051) at 12 weeks and a significant increase in WB on the LH at 15 and 24 weeks. In contrast, the MIUChon treatment group exhibited a gradual increase in WB on the RH with induced OA following MIUChon treatment, with no significant difference in WB between the two hindlimbs (Fig. 5B).

To further verify the efficacy of MIUChon in alleviating OA symptoms and improving mobility, the guarding index (GI)—a quantitative measure of an animal’s tendency to protect a painful limb or joint by bearing less weight—was assessed (Fig. 5C). The difference in WB between the two hindlimbs of each group before OA induction was set to 1 for comparison across different time points (Fig. 5C).

The diagonal weight-bearing distribution (DWBD) of four animals each from the vehicle control group and the MIUChon treatment group was calculated (table S4). Using the data from table S4, changes in individual DWBD (ΔDWBD) at each time point were summarized (table S5). The ΔDWBD values were compared between the vehicle control group and the MIUChon treatment group at each time point. The MIUChon treatment group showed statistically significant changes in ΔDWBD at 12 and 18 weeks (Fig. 5D).

A comprehensive comparison of ΔDWBD at each time point also showed statistically significant improvements in the MIUChon treatment group (Fig. 5E). Comparing the mean values of ΔDWBD for each time point between the vehicle control group and the MIUChon treatment group, the overall mean ΔDWBD value in the MIUChon treatment group (Δ6.925%) was significantly higher—5.3 times higher—compared to the overall mean value (Δ1.3075%) of the vehicle control group.

## DISCUSSION

The development of effective and accessible therapies for cartilage regeneration shows great potential for patients with diseases that affect the joints. In this study, we developed an efficient and reproducible manufacturing process for generating clinical-grade MIUChon, micro-chondrogenic pellets that can be delivered through IA injections. The present study demonstrates the therapeutic efficacy of MIUChons in treating OA, validating both a clinical-grade manufacturing platform and potent in vivo cartilage regeneration.

OA is a progressive disease characterized by degraded cartilage in the joint due to physical injury or chronic inflammation. Although several attempts have been made, there are currently no specific clinical interventions that ensure successful treatment (*31*). In the early years of cell-based therapy, adult stem cells such as MSCs or isolated primary chondrocytes were extensively used in cartilage repair and regeneration (*32*–*35*). Showing primarily paracrine effects, these attempted therapeutics demonstrated efficient modulatory effects, but failed to achieve regeneration (*36*, *37*). In contrast, pluripotent stem cell–based treatments represent a next-generation cell therapy technology and are widely applied to various diseases.

We developed a workflow to manufacture iPSC-derived MIUChons from healthy cord blood mononuclear cell (CBMC)-derived iPSCs. Because of their progenitor and proliferative characteristics, CBMCs isolated from healthy donors were considered an acceptable source for generating clinical-grade iPSCs, making them a suitable choice for clinical applications (*34*). In addition, clinical-grade CBMC-derived hiPSC lines confirmed the prochondrogenic characteristics of the cell lines by successfully generating chondrogenic macropellets in vitro (*38*). The generated MIUChons showed a significant, time-dependent increase in key markers of chondrogenesis, along with abundant ECM secretion, a hallmark characteristic of chondrocytes.

To elucidate the differentiation process from iPSCs into MIUChons and investigate whether MIUChons have prochondrogenic characteristics, scRNA-seq was performed on iPSCs (labeled as MCB), mesenchymal-like OGCs at the fourth passage (OGC_P4), and MIUChons differentiated at four time points [days 3 (D3), 7 (D7), 13 (D13), and 14 (D14)]. Through UMAP clustering analysis, we confirmed cellular heterogeneity in iPSCs from the MCB, progenitor OGCs, and MIUChons. High similarity in gene expression data was identified between MIUChon-D3 and -D7, as well as between MIUChon-D13 and -D14, resulting in each pair belonging to the same cluster. However, there was noticeable heterogeneity between MIUChon-D3/D7 and MIUChon-D13/D14. Specifically, MIUChon-D3/D7 formed three major clusters (CL 0, 1, and 3), whereas MIUChon-D13/D14 formed a distinct major cluster (CL 2). This result illustrates the difference between early-stage (MIUChon-D3/D7) and mature-stage (MIUChon-D13/D14) MIUChons. On day 13 of differentiation, the mature cells were likely to have reached a uniform chondrospheroid population. The expression of marker genes related to chondrocyte and/or cartilage development, such as *COL2A1*, *COMP*, *MATN4*, *EGR1*, *ERG*, and *SOX6*, was evaluated in both early-stage (clusters 0, 1, and 3) and mature-stage (cluster 2) MIUChons. Pronounced expression of these genes was observed in mature MIUChon cells. In addition, genes such as *TGFB1/2*, *TIMP1/2/3*, *ADAMTS6*, and *FBLN5*, which are essential for positively regulating cartilage development, were highly expressed from OGCs to MIUChons (*39*–*41*). These results suggest that mature MIUChons are not merely chondrocyte aggregates but rather chondrospheroids that play a pivotal role in cartilage development, particularly by enhancing functions related to cartilage ECM organization.

The establishment of proper OA animal models to validate in vivo efficacy was a crucial aspect of this study. Initially, MIA rat models and ACLT rabbit models were used to confirm the in vivo efficacy of MIUChons in a proof-of-concept study. We recognized that MIA rat models and ACLT rabbit models do not fully recapitulate OA pathophysiology; however, these models demonstrated improved clinical effects in cartilage defects, showing the potential efficacy of the generated MIUChons. Although we did not perform a complete dose-response study for the injected spheroids, we aimed to inject 4700 units of MIUChon in humans, which are equivalent to the cell numbers commonly used in previous clinical applications of MSCs delivered by IA injections (*42*, *43*). The injected MIUChon dosage was calculated based on the human-equivalent dose and resulted in the injection of a small amount of MIUChons in rats (30 to 40 MIUChon units) and rabbits (450 to 500 MIUChon units). This dosage limitation was believed to hinder the ability to confirm efficacy, prompting us to develop a larger animal model for further validation. The above considerations highlight why the small-joint animal models provided only limited insight into MIUChon’s regenerative capacity. The MIA-induced OA rat model, while useful for initial screening, causes rapid chondrocyte death and loss of ECM within weeks (*44*), making it challenging to detect restored aggrecan content by the time of analysis. Aggrecan immunostaining in MIUChon-treated rat cartilage remained similar to controls, likely because MIA depletes aggrecan within 1 to 2 weeks and the rat knee’s limited synovial volume (<0.1 ml) severely restricts the contact area and engraftment of 150 to 200 μm MIUChon. Consequently, even though treated rats showed a thicker cartilage layer and the presence of human MIUChon cells in the joint, the robust cartilage matrix integration seen in larger models could not be fully realized in this small model. We therefore transitioned to large-joint OA models (ACLT in rabbits and combined ACLT + MMx in minipigs and beagle dogs) to better emulate the clinical scenario of advanced cartilage defects and to provide a sufficient IA space for MIUChon delivery and integration. Notably, larger animals have thicker cartilage and more human-like joint biomechanics, which allow for more accurate evaluation of cartilage repair. In addition, to eliminate any confounding effect of injection mechanics, we ensured that the vehicle (cell-free injection solution) was formulated to match the viscosity of the MIUChon suspension.

During the process of establishing beagle and minipig OA models, we aimed to generate a more OA-like cartilage defect that would better mimic patients with stage 2 to 3 OA. Compared to ACLT or MIA models, the combination of ACLT and MMx produced more significant and severe cartilage defects, enabling us to confirm the efficacy and potential for direct regeneration of MIUChons. RNA-seq of cartilage tissue in the treated animals suggested that MIUChon treatment in OA leads to a reduction in the immune response, decreased ECM degradation, and the promotion of cartilage development. Notably, the reduction in ER overload response in the MIUChon-treated group indicates that the synthesis and folding of cartilage-related proteins functioned normally, potentially leading to decreased cell death associated with these processes (*45*). A primary concern in applying pluripotent stem cells is the risk of residual tumorigenicity. Possible tumorigenicity was assessed in the original iPSCs, progenitor cells, and generated MIUChons by confirming TRA-1-60 expression and performing teratoma assays. We also evaluated the genetic stability and safety of MIUChons by analyzing STRs, somatic mutations, and CNVs. STR analysis was conducted for cell line authentication, amplifying a sex-determining marker, amelogenin, and 15 STR loci. The results demonstrated genetic homogeneity among the MCB, WCB, and MIUChons. Although a significant number of somatic variants (an average of 3291 across three replicates) were detected in MIUChons compared to the WCB, the frequency of somatic coding variants was relatively low. Among these somatic coding variants, two were predicted to damage protein structure, as determined by PolyPhen2 and SIFT. However, structural predictions using Missense3D (*46*) indicated that these variants would not affect the structural integrity of their respective proteins. All identified somatic coding variants were deemed nonpathogenic based on searches in the ClinVar and COSMIC databases, which assess the clinical significance of variants and their contribution to tumor risks, respectively.

Another consideration for iPSC-based cell therapeutics is immunity. A large proportion of cartilage consists of ECM, which surrounds chondrocytes and shields them from external factors or immune cells. This characteristic makes cartilage an immune-privileged tissue, making engineered cartilage-like tissues more feasible as alternative off-the-shelf grafts for transplantation (*47*). Allogeneic cartilage has been clinically used without matching HLA types or immunosuppressants (*48*–*50*). A recent study by Abe *et al.* (*51*) explored the immune response to allogeneic iPSC-derived chondrogenic tissues in primate osteochondral defect models. The implanted cartilage organoids integrated with the host native articular cartilage, indicating that chondral defects could be a definitive indication for treatment. In our study, although direct confirmation of cartilage regeneration by injected MIUChons was not achieved, increased cartilage volume and reduced defect areas suggest that MIUChons facilitated recovery or prevented disease progression. Future studies are required to investigate whether MIUChons have a direct role in healing injuries or whether their effects are mediated through paracrine effects.

Mounting evidence from our multimodel experiments supports a dual MoA for MIUChon in repairing cartilage. First, MIUChon-derived chondrocytes can directly contribute to tissue regeneration by integrating into defect sites and producing cartilage matrix. In a rabbit osteochondral defect model, implanted MIUChons survived and exhibited lacunae-like morphologies with intense Safranin O staining, indicating new hyaline-like cartilage formation by the graft. Similarly, in the rat MIA model, we detected human nuclei–positive chondrocytes within the treated cartilage, demonstrating that MIUChon cells can engraft and persist in vivo. Consistently, cross-species molecular analyses demonstrated that human molecules delivered by MIUChon persisted within the joint environment and contributed to regeneration, as shown by the detection of human-specific proteins in rabbits and human-specific transcripts in minipigs enriched in ECM remodeling and cytoskeleton organization. Together with our proteomic profiling of rabbit cartilage (fig. S16), which highlighted pathways related to engraftment, ECM restoration, and chondrogenic activation, these findings indicate that direct engraftment of human-derived cells enables the persistence of functional human molecules that contribute structurally and transcriptionally to cartilage repair. Moreover, previous studies have shown that iPSC-derived cartilage implants can structurally integrate into host tissue without tumor formation (*51*, *52*). Second, MIUChons exert paracrine immunomodulatory effects that create a proregenerative joint milieu. In our large-animal OA models, MIUChon treatment elevated regenerative growth factors (e.g., TGF-β3 and BMP-2) and anti-inflammatory cytokines (e.g., IL-4), while suppressing proinflammatory cytokines such as IL-6, TNF-α, IL-17A, and CXCL10. Consistently, MIUChon-treated cartilage tissues showed down-regulation of catabolic and stress-related genes alongside up-regulation of cartilage development pathways. This immunomodulatory secretome likely promotes an environment conducive to cartilage repair and protects resident chondrocytes from further damage. Together, these findings support a dual MoA whereby MIUChon both directly regenerates cartilage and indirectly stimulates repair through trophic and immunomodulatory signals. This dual mechanism is in line with the current understanding of stem cell therapies for OA, wherein transplanted cells can serve as building blocks for new tissue while simultaneously orchestrating the host’s repair response.

Our proteomic, transcriptomic, and histological analyses indicate that MIUChon engrafts and persists within the joints of rabbit and minipig models, contributing to ECM remodeling and proregenerative immune signaling. However, because these findings were obtained in a xenogeneic setting in which human cells were administered into nonhuman hosts, the immunological and stromal milieu including HLA and minor-antigen mismatches, complement and NK-cell activation, and cytokine-mediated regulation of HLA expression may differ from that encountered following allogeneic IA administration in humans. Although the relative immune privilege of cartilage and the successful engraftment observed in immunocompetent large-animal models without immunosuppression partially mitigate these concerns, and despite feasibility suggested by prior studies of allogeneic cartilage and PSC-derived grafts, we cannot fully exclude the possibility that the extent and durability of engraftment, as well as the magnitude of paracrine immunomodulatory effects, will differ in human allogeneic settings. Together, our findings support a dual MoA whereby MIUChon both directly integrates into cartilage and indirectly promotes repair through trophic and immunomodulatory signaling, establishing the reproducibility and stability of these mechanisms in allogeneic human joints remains a critical prerequisite for clinical translation.

## MATERIALS AND METHODS

### iPSC culture

Following procedures detailed in our prior report (*53*), the clinical-grade hiPSC lines used in this study were derived from CBMCs. CBMCs, which are highly proliferative and contain a high CD34^+^ population, were stored in the Catholic Hematopoietic Stem Cell Bank. The generated iPSCs were passaged, and research cell banks, MCBs, and WCBs were established. These iPSC banks were characterized and tested for purity, focusing on the loss of reprogramming factors, sterility, and genomic stability, including G-band karyotyping and short-tandem repeat identity (table S1).

### Chondrogenic differentiation

MIUChons were generated following protocols established in previous studies, with modifications to optimize clinical compatibility (*17*, *54*). Chondrogenic differentiation was induced by activating TGFs or bone morphogenetic proteins in mesenchymal-like cells derived from stem cells. Embryoid bodies (EBs) were generated to promote mesodermal lineage differentiation in iPSCs.

EBs were maintained in CTS Essential 8 medium (Thermo Fisher Scientific) for 5 days, followed by E7 medium for an additional 5 days at 5% CO_2_ and 37°C. Subsequently, EBs were collected and placed in OG induction medium formulated with Dulbecco’s Modified Eagle’s Medium (Thermo Fisher Scientific), 10% fetal bovine serum (Thermo Fisher Scientific), and 0.004% Recombinant human FGF basic (FGF2, PeproTech). The EBs were then seeded onto laminin-coated plates to induce OG proliferation and later transferred to fibronectin-coated dishes for up to four passages.

OGCs were counted, and 3 × 10^6^ cells were seeded in AggreWell plates (STEMCELL Technologies). The original medium was exchanged for a chondrogenic differentiation medium containing recombinant human BMP2 (50 ng/ml; Gibco) and TGFβ3 (50 ng/ml; Gibco). Subsequently, cells were centrifuged in microwells at 750*g* for 5 min to form chondrogenic pellets, which were then maintained for 14 days.

The final MIUChons were characterized based on morphology, cell count, viability, impurity, sterility, and the expression of CD44 and COL2A1 (>70%). Loss of iPSC markers was also confirmed (table S2). A total of 13 batches were manufactured, including 7 preclinical batches and 6 quality verification batches.

### Quantitative real-time PCR

Samples were collected and immediately snap frozen in liquid nitrogen. The frozen pellets were then ground using a pestle and treated with TRIzol reagent (Thermo Fisher Scientific) to isolate RNA. The extracted RNA was used to synthesize cDNA with the RevertAid First Strand cDNA Synthesis Kit (Thermo Fisher Scientific). Quantitative real-time polymerase chain reaction (PCR) was subsequently carried out on a LightCycler 480 Instrument II (Roche, Basel, Switzerland), and the relative expression of each target gene was determined following normalization.

### scRNA-seq and bioinformatics analysis

Single-cell suspensions with at least 90% viability were loaded onto the 10x Chromium Controller using Chromium Next GEM Single Cell 3′ Reagent Kits v3.1 (10× Genomics), following the manufacturer’s instructions. Approximately 10,000 cells were encapsulated in nanoliter-scale Gel Beads-in-Emulsion. Single-cell 3′ mRNA libraries were generated via reverse transcription, cDNA amplification, fragmentation, and adaptor ligation, followed by dual-index PCR. Library quality was evaluated using a Bioanalyzer (Agilent Technologies). Sequencing was performed on an Illumina NovaSeq 6000 system with the following cycle setup: read 1 (28 cycles), i7 index (10 cycles), i5 index (10 cycles), and read 2 (90 cycles). Each library yielded an average of approximately 398.4 million reads.

Paired-end reads were processed using Cell Ranger (10x Genomics, v7.0.0), which handled demultiplexing, barcode filtering, gene quantification, and annotation. Reads were aligned to the human GRCh38-2020-A reference genome. Any barcodes below the 10% threshold of the 99th percentile for total unique molecular identifier (UMI) counts—potentially corresponding to empty droplets—were discarded. After quality control, gene barcode matrices documenting UMI counts for each cell and gene were produced.

Subsequent analyses were performed using the Seurat R package (v4.3.0.1) (*47*). Cells expressing fewer than 450 genes, more than 7500 genes, or more than 8% mitochondrial genes were excluded. Gene expression data were normalized with SCTransform, and confounding factors such as cell cycle phase (S and G_2_-M scores) and mitochondrial gene content were regressed out using the vars.to.regress argument.

For unsupervised clustering, highly variable genes were identified via the FindVariableFeatures function, and significant principal components were computed for dimensionality reduction. Clustering was then conducted using the FindNeighbors and FindClusters functions with a resolution of 0.3, and the resulting clusters were visualized using UMAP.

Following cluster determination, DEGs were identified using the FindAllMarkers function. DEGs were deemed significant if they satisfied *P* < 0.001, an absolute log_2_ fold change greater than 0.584, and a difference in expression frequency (pct.1 − pct.2) exceeding 0.2. Functional enrichment analysis was conducted for the significant DEGs using the DAVID Gene Functional Classification Tool (v6.8), applying an expression alnalysis systematic explorer (EASE) score cutoff of <0.01.

### Sample preparation for histological analysis

ChondroPellets were collected in 1.5-ml Eppendorf tubes, rinsed with Dulbecco’s phosphate-buffered saline (Thermo Fisher Scientific), and fixed in 4% paraformaldehyde (T&I) for 2 hours at room temperature (RT). The fixed pellets were then dehydrated through a graded ethanol series, cleared by incubations in ethanol (DuksanCNP)–xylene (Daejung) mixtures (culminating with 100% xylene), and lastly infiltrated with molten paraffin overnight at 65°C. For joint tissues from rats, rabbits, and beagles, gross examinations were performed after euthanasia. Large tissues were ground to achieve dimensions of less than 1 cm by 1 cm by 1 cm. Beagle joint tissues were separated into tibial, femoral, lateral, and medial condylar regions. All tissues were fixed in 4% paraformaldehyde for 24 hours at RT, washed with running tap water, and decalcified with a decalcifying solution (Sigma-Aldrich). Rat and rabbit samples were decalcified for 6 hours, whereas beagle samples required 72 hours. Samples were rinsed with running tap water, cleared through ethanol-xylene incubations, and embedded in paraffin overnight. Paraffin blocks were sectioned at 7 μm thickness using a microtome for histological analysis.

### Animals

Both surgical and nonsurgical interventions involving animals were approved by the Animal Studies Committee at the School of Medicine, Catholic University of Korea. All procedures complied with the Laboratory Animal Welfare Act, the Guidelines and Policies for Rodent Survival Surgery, and the ARRIVE (Animal Research: Reporting of In Vivo Experiments) guidelines. Euthanasia was conducted using CO_2_, following the American Veterinary Medical Association (AVMA) Guidelines for Euthanasia of Animals (2020 edition). The animal experiments were approved by the Institutional Animal Care and Use Committee (IACUC) of School of Medicine, The Catholic University of Korea (approval numbers: CUMS-2016-0291-02, CUMS-2017-0144-05, and CUMS-2019-0281-07).

### OA induction and MIUChon injections

OA was induced in the RH of Sprague-Dawley rats (Orient Bio, Korea), via IA injection of MIA. Four weeks after OA induction, MIUChon was administered as a single dose, and the animals were euthanized at 12 weeks for gross histological and histopathological analyses.

For the rabbit experiments, OA was induced in 8-month-old New Zealand White rabbits (Orient Bio, Korea) by performing ACLT on the right knee joint. Four weeks postinduction, MIUChons were administered via IA injections, and its efficacy was evaluated at 8 weeks following a 4-week observation period, with an additional evaluation conducted at 12 weeks after an extended 8-week observation period.

For the beagle studies, OA was induced in the LH of 15-month-old beagle dogs (Woojung BSC, Korea) through ACLT. MIUChon was administered 4 weeks postinduction, and efficacy was assessed at 14 weeks through animal sacrifice.

In the minipig study, OA was induced in 30-month-old minipigs (APURES, Korea) using ACLT combined with MMx. MIUChon was administered 8 weeks postinduction, and the animals were monitored for 24 weeks. Body weight changes were recorded three times: before OA induction, on the day of MIUChon administration, and at necropsy, to observe any drug-related effects. Behavioral assessments were conducted using the Zebris CanidGait system, which measured weight distribution across the limbs as the animals walked across a sensor-embedded rubber pad (3000 mm by 600 mm). The WB data were analyzed using indices such as the SI, diagonal area summation index (ASI), and GI for a comprehensive evaluation. The “vehicle” is the identical injection solution without MIUChon (0.9% saline).

### Behavioral assessment in minipigs

Gait assessment was conducted using a gait analyzer (CanidGait, Zebris) with a 3000 mm by 600 mm rubber pad embedded with weight load detection sensors. Minipigs with surgically induced OA (ACLT + MMx) were allowed to walk quadrupedally across the pad at a controlled trotting pace. WB (%) was recorded for each limb at time points 0, 8, 12, 15, 18, and 24 weeks postinduction. On the basis of these WB data, several indices were calculated to evaluate gait asymmetry and compensatory weight redistribution. The SI was calculated as [(WBL − WBR)/(WBL + WBR) × 0.5] × 100, reflecting weight-bearing asymmetry between the LH and RH. The CWBD was defined as WB_RH_/(WB_LH_ + WB_RH_) × 100, to assess compensatory redistribution of weight between the OA-affected RH and the contralateral limb. The GI was determined by calculating the difference in WB between the healthy and OA-affected hindlimbs, with baseline values before OA induction set to 1, and relative changes compared across subsequent time points. In addition, the DWBD was calculated as WB_RH_/(WB_LF_ + WB_RH_) × 100, and the change in DWBD (ΔDWBD) was derived from differences between consecutive time points for each animal, reflecting longitudinal alterations in diagonal gait patterns. All parameters were analyzed both at the individual and group levels, and statistical significance was determined using independent *t* tests or nonparametric Mann-Whitney tests depending on data normality.

### Merge and regeneration test of MIUChon

New Zealand White Rabbits were used for this experiment. Rabbits were 8 months old when the defect was developed. Rabbits were anesthetized, and a defect was created in articular cartilage of the trochlear groove of the distal femur using a microdrill. The generated osteochondral defect had a diameter of 3.0 mm and a depth of 1.5 mm. After the surgery, the defects were washed with normal saline. MIUChons were transplanted immediately after generating the defect. After 1 week, rabbits were euthanized for histological analysis.

### Histological staining of the cartilage tissue

Slides were placed on a 60°C heating stage for at least 10 min and then immediately deparaffinized in two consecutive 100% xylene baths. Rehydration was carried out by passing the slides through a series of decreasing ethanol concentrations, followed by a rinse under running tap water before staining.

For safranin O staining, slides were immersed in Weigert’s hematoxylin (Sigma-Aldrich) for 1 min at RT, rinsed in tap water, stained with 0.001% fast green (Sigma-Aldrich) for 5 min at RT, and washed with 1% acetic acid (Sigma-Aldrich). Next, they were incubated in 0.1% safranin O (Sigma-Aldrich) for 10 min.

For Alcian blue staining, slides were soaked in 1% Alcian blue (Sigma-Aldrich) for 30 min at RT, rinsed under running water, and counterstained with nuclear fast red solution. For toluidine blue staining, rehydrated slides were immersed in 0.04% toluidine blue (Sigma-Aldrich) for 10 min, washed under running tap water, and dried thoroughly for 10 min.

Upon completion of each staining protocol, slides underwent dehydration in a series of ethanol baths, were cleared in two fresh 100% xylene baths, and were lastly mounted using VectaMount Permanent Mounting Medium (Vector Laboratories).

### Immunohistochemistry

Slides were initially warmed at 60°C for at least 10 min and then immersed in two consecutive 100% xylene baths to remove paraffin. Rehydration was performed with a series of decreasing ethanol concentrations, followed by a rinse under running tap water. Endogenous peroxidase was quenched using 3% hydrogen peroxide (Sigma-Aldrich), and the slides were washed and blocked in tris-buffered saline (TBS) containing 10% normal serum (Vector Laboratories).

Primary antibodies for type I collagen (1/200; Abcam) and type II collagen (1/100; Abcam) were prepared in the blocking solution. The slides were incubated with these antibodies at 37°C for 30 min, then washed in TBS supplemented with 0.1% Tween-20. Next, secondary antibodies (1/200; Vector Laboratories) diluted in blocking buffer were applied for 40 min at RT.

Following another wash, ABC reagent (Vector Laboratories) was added for 30 min, and the slides were again rinsed before treatment with 3,3′-diaminobenzidine (DAB, Vector Laboratories) for 1 min at RT, closely monitored for color development. Slides were counterstained with Mayer’s hematoxylin (Sigma-Aldrich) for 1 min, dehydrated in a graded ethanol series, cleared in 100% xylene, and lastly mounted.

### Immunofluorescence staining

Slides were initially warmed at 60°C for at least 10 min and then immersed in two consecutive 100% xylene baths to remove paraffin. Rehydration was performed with a series of decreasing ethanol concentrations, followed by a rinse under running tap water. Endogenous peroxidase was quenched using 3% hydrogen peroxide (Sigma-Aldrich), and the slides were washed and blocked in phosphate-buffered saline (PBS) containing 2% bovine serum albumin (BSA; Vector Laboratories) for 1 hour at RT. Primary antibodies were diluted with a mixture of PBS plus BSA (PBA) and incubated at RT for 2 hours. The following dilution ratios were used: anti-Nuclei (1/200; Abcam, ab254080), anti–type II collagen (1/200, Abcam), and anti–TRA-1-60 (1/200, EMD Millipore). After washing the slides, Alexa Fluor 594 (1/400, Life Technologies)– and Alexa Fluor 488 (1/400, Life Technologies)–conjugated secondary antibodies were diluted in PBA and incubated at RT in the dark for 1 hour. Secondary antibody was washed followed by 4′,6-diamidino-2-phenylindole (DAPI) staining for 10 min at RT. Slides were washed and mounted using antifade mounting reagent (Thermo Fisher Scientific). The stained slides were observed under a high-powered magnification using a confocal microscope (Carl Zeiss).

### Study design

This study aimed to develop an iPSC-based therapy for OA. We developed clinical-grade chondrogenic spheroids termed “MIUChon” from HLA-homozygous clinical-grade hiPSCs. The generated chondrogenic micropellets were validated and tested for safety and efficacy in preclinical animal models. Three to five different clones were used for all in vitro experiments to validate the MIUChons. In addition, rat experiments were repeated at least three times.

### Statistical analysis

Statistical analyses for the in vitro functional validation of the MIUChons were performed using GraphPad Prism (GraphPad Software).
