# Supplementary material for: Clinical-grade iPSC-derived chondrogenic micropellets for treating advanced cartilage defects
Source: Sci Adv. 2025 Dec 17;11(51):eadw4911. doi: 10.1126/sciadv.adw4911 (PMC12710702; doi:10.1126/sciadv.adw4911)
Supplement: Supplementary file 1 — Figs. S1 to S17 Tables S1 to S6 [file sciadv.adw4911_sm.pdf]

Science Advances
AAAS

Supplementary Materials for

Clinical-grade iPSC-derived chondrogenic micropellets for treating advanced cartilage defects

Yoojun Nam *et al.*

Corresponding author: Yeri Alice Rim, llyerill0114@gmail.com; Ji Hyeon Ju, juji@catholic.ac.kr

Sci. Adv. **11**, eadw4911 (2025)
DOI: 10.1126/sciadv.adw4911

This PDF file includes:

Figs. S1 to S17
Tables S1 to S6

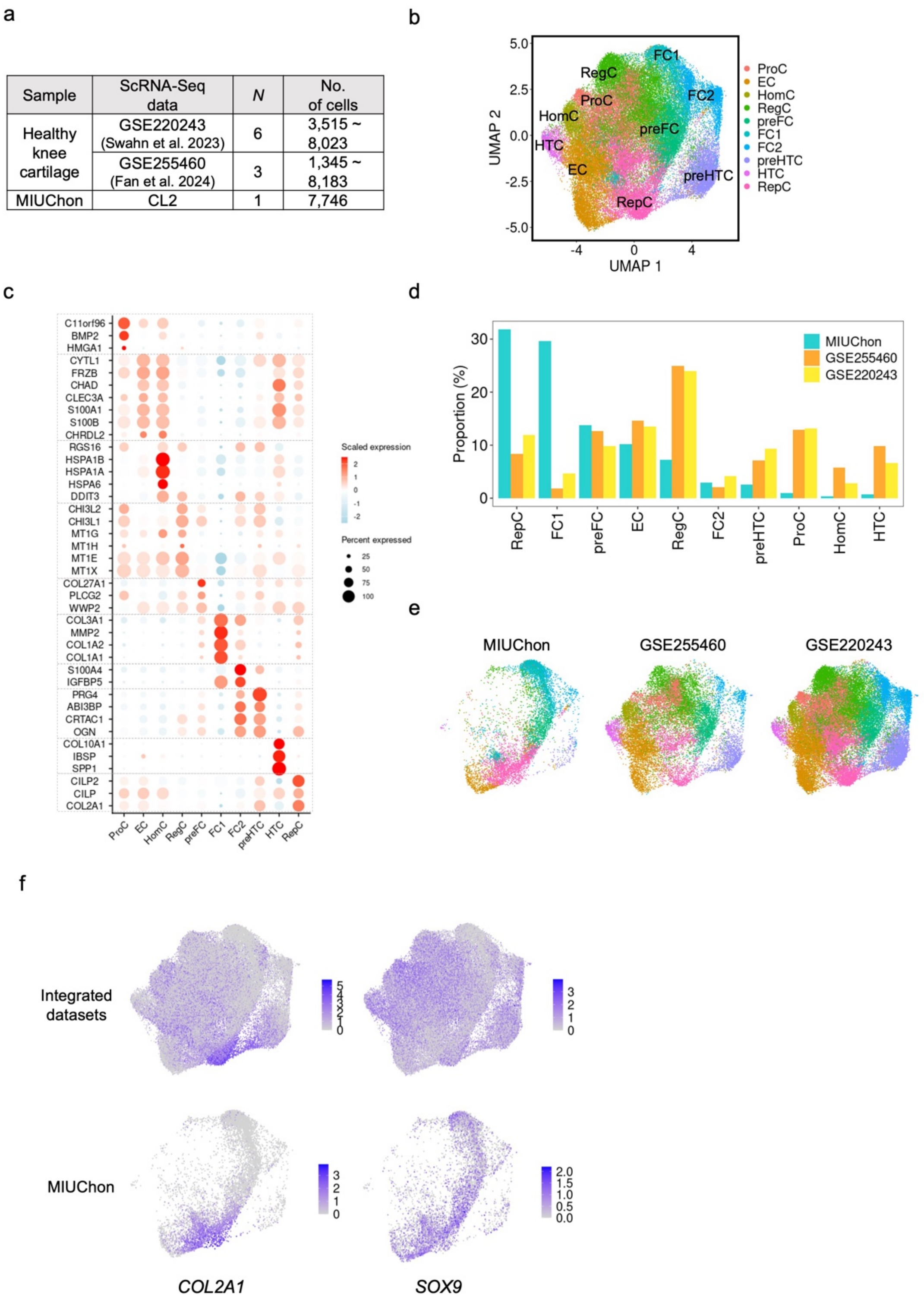

Supplementary Figure 1. Comparative single-cell transcriptomic analysis of MIUChon-derived chondrocytes and native human cartilage. a) Summary of the single-cell RNA-seq datasets used in the analysis. Publicly available datasets from healthy human articular cartilage [GSE220243 and GSE255460] were integrated with MIUChon CL2 data. Datasets were normalized using SCTransform and integrated using the *IntegrateLayers* function in Seurat with the CCA integration method to mitigate batch effects. **b)** UMAP plot of integrated data showing ten distinct chondrocyte subpopulations classified based on transcriptomic profiles: proliferation

chondrocyte (ProC), effector chondrocyte (EC), homeostatic chondrocyte (HomC), regulatory chondrocyte (RegC), reparative chondrocyte (RepC), fibrocartilage chondrocyte 1 (FC1), fibrocartilage chondrocyte 2 (FC2), pre-fibrocartilage chondrocyte (preFC), pre-hypertrophic chondrocyte (preHTC), and hypertrophic chondrocyte (HTC). **c)** Dot plot showing the expression of representative marker genes used to define each chondrocyte subpopulation. Dot size indicates the percentage of cells expressing the gene, and color intensity reflects the scaled expression level. **d)** Proportions of each chondrocyte subtype across the three datasets: MIUChon (cyan), GSE255460 (orange), and GSE220243 (yellow). **e)** UMAP plots showing the distribution of chondrocyte subtypes in each individual dataset. f) Feature plots showing the expression patterns of cartilage-specific marker genes *COL2A1* and *SOX9* in the integrated dataset (top) and the MIUChon dataset alone (bottom). Both genes are highly expressed in chondrocyte subtypes across MIUChon and native cartilage datasets, supporting the chondrogenic identity of MIUChon-derived cells. Color intensity indicates normalized expression levels.

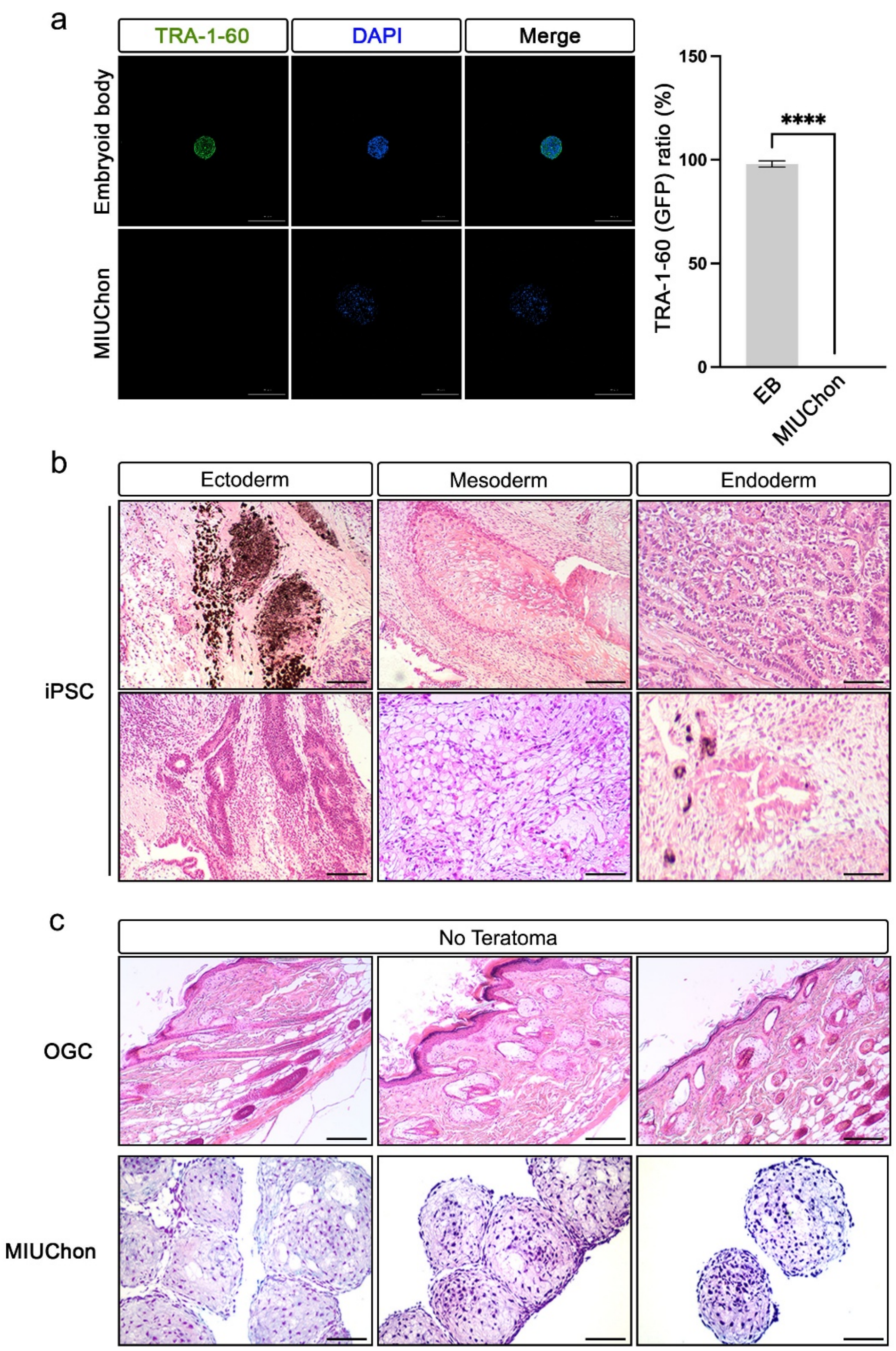

Supplementary Figure 2. Tumorigenicity study of MIUChon. **a**) TRA-1-60 expression in MIUChon and iPSC-derived embryoid bodies (EBs) as a positive control. **b**) Histological analysis of the iPSC-derived tumor. **c**) Histological analysis of outgrowth cells (OGCs) and MIUChons. Scale bars indicate 200 μm.

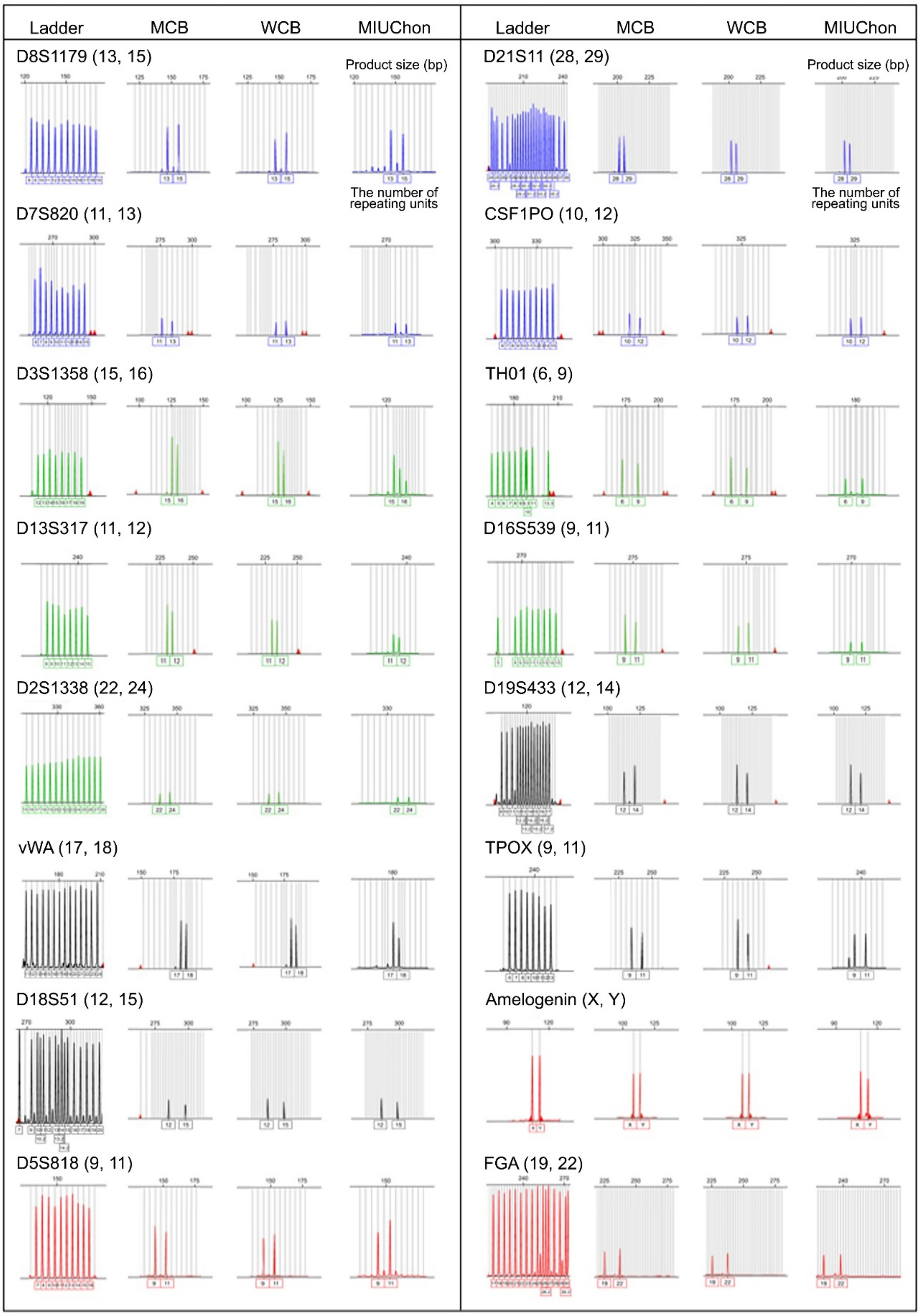

Supplementary Figure 3. Confirmation of genetic homogeneity among MCB, WCB, and MIUChon at the STR loci.

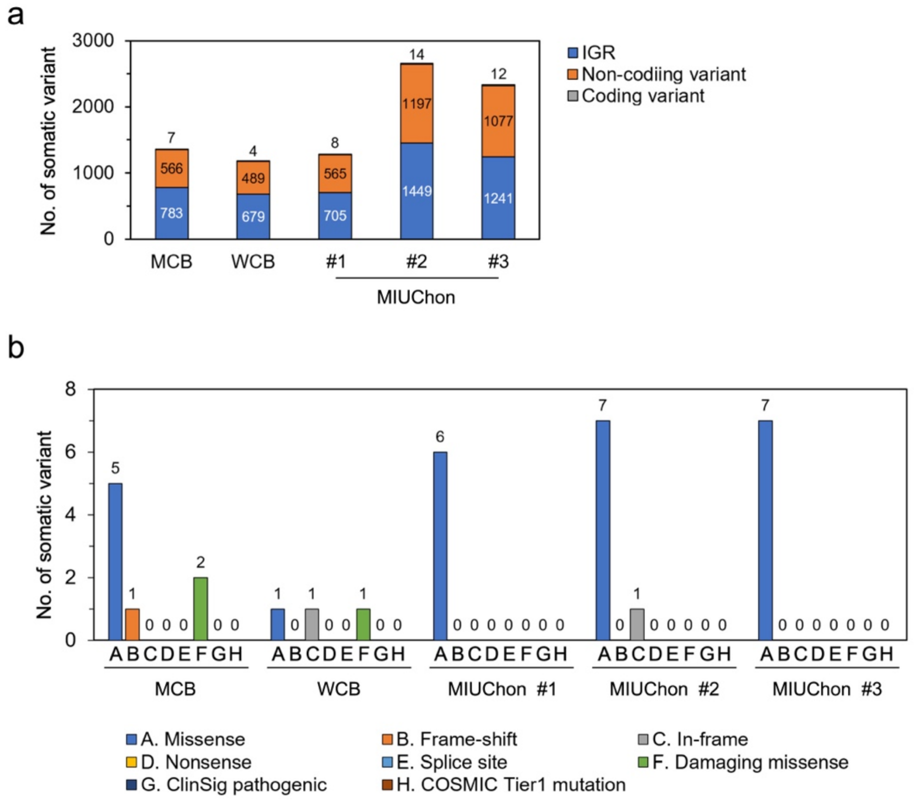

Supplementary Figure 4. Pathogenic variant identification through somatic coding variant alaysis in MCB, WCB, and MIUChon. **a**) Frequency of somatic varients. **b**) Further analysis of the somatic coding varients.

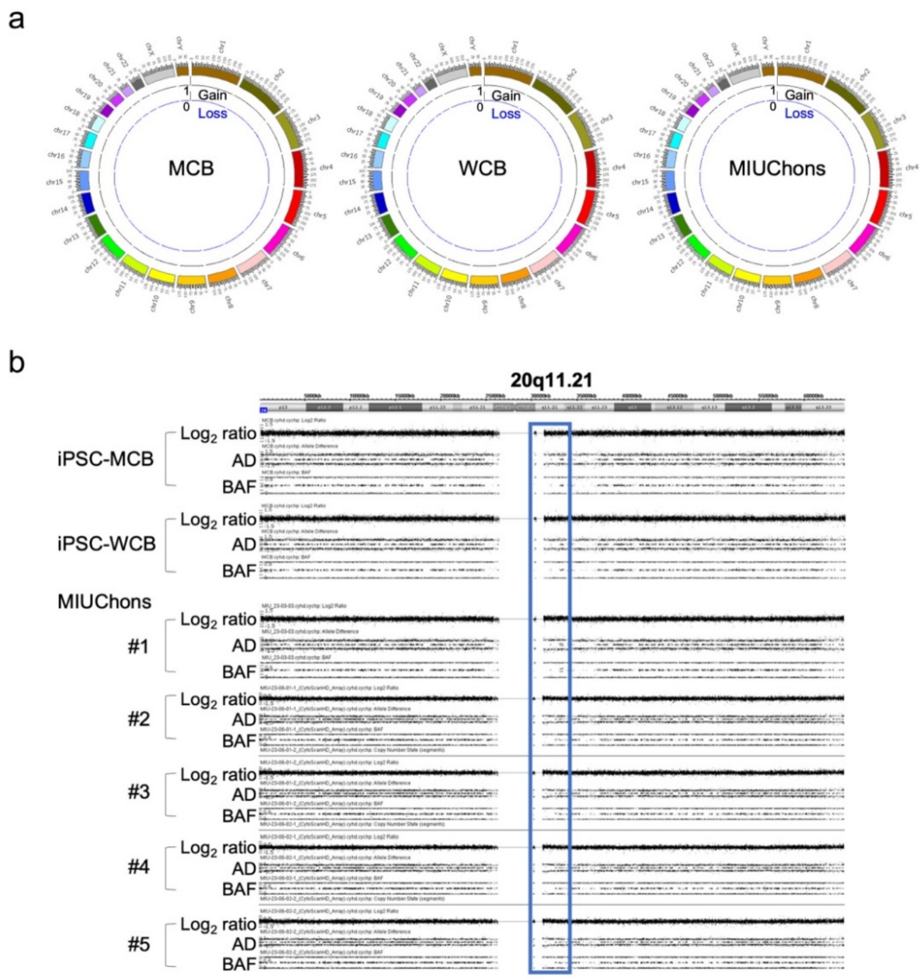

Supplementary Figure 5. CNV analysis in MCB, WCB, and MIUChon. **a**) No CNVs were detected in the whole genomes of MCB, WCB, and MIUChon. **b**) The absent of a recurrent genomic aberration, 20q11.21, in human pluripotent stem cells was confirmed.

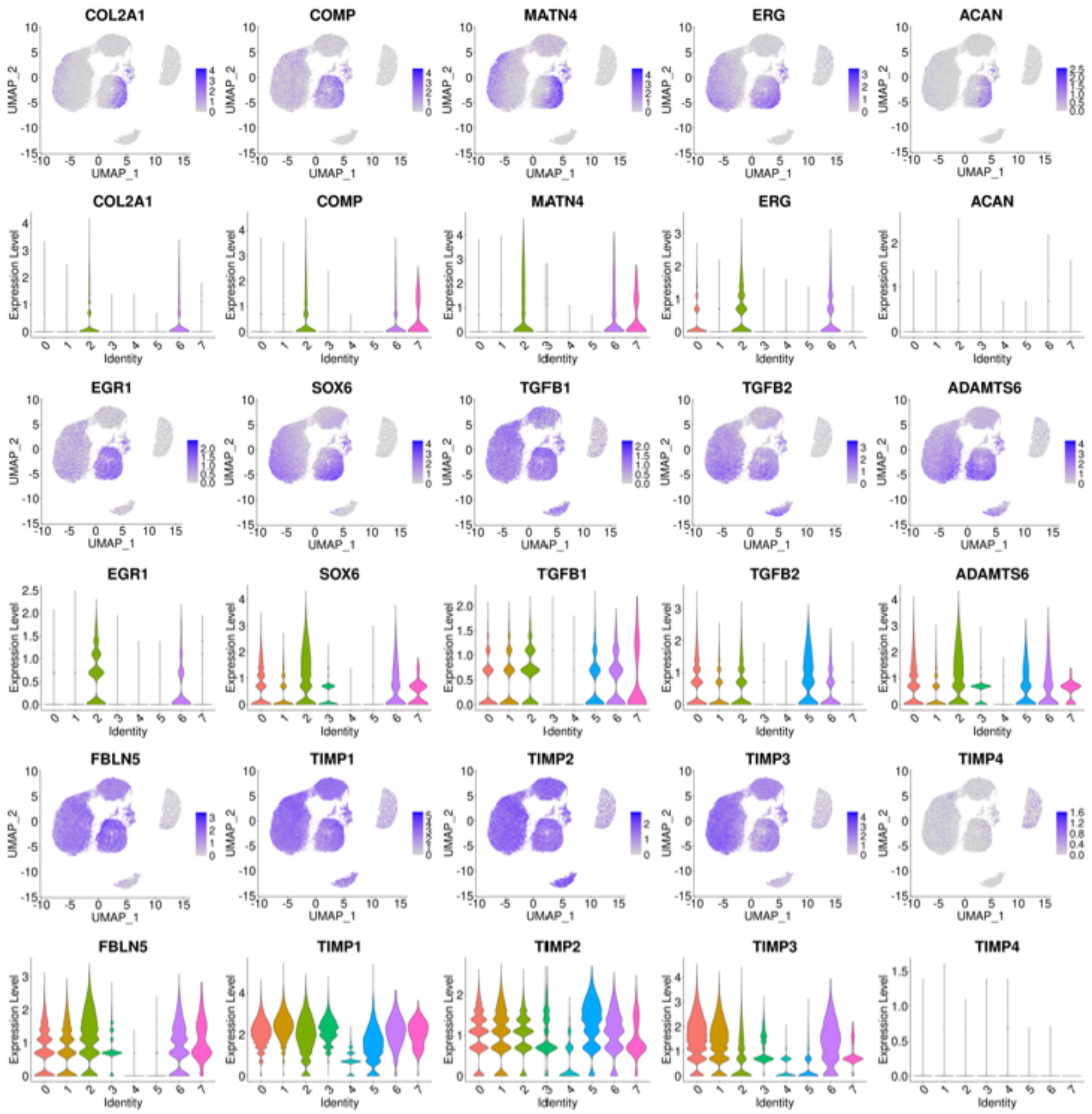

Supplementary Figure 6. Confirmation of ECM and cartilage-related expression in OGC and MIUChon clusters.

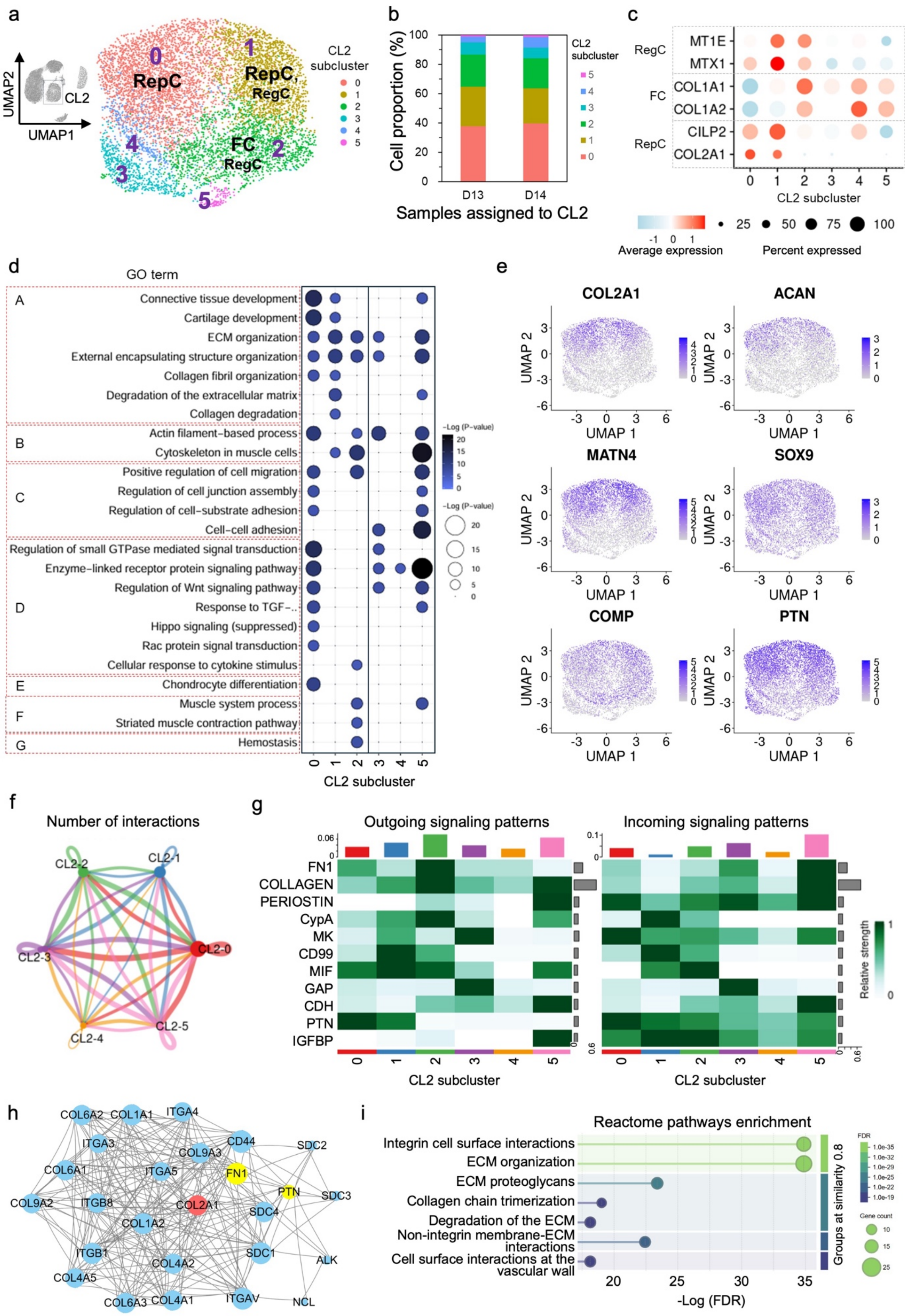

Supplementary Figure 7. Single-cell characterization and cell–cell communication network of mature MIUChon cluster CL2. a) Subclustering of the mature-stage MIUChon cluster CL2 based on UMAP analysis.

The six subclusters within CL2 are visualized using UMAP. Clusters 0, 1, and 2 are predicted to correspond to reparative chondrocytes (RepC), a mixture of RepC and regulatory chondrocytes (RegC), and a mixture of fibrocartilage chondrocytes (FC) and RegC, respectively, based on Supplementary Figure 14c. **b)** Proportions of cells in each CL2 subcluster from the CL2-associated samples (D13 and D14). **c)** Expression of marker genes characteristic of cell types such as RegC, FC, and RepC. **d)** Gene ontology (GO) enrichment analysis for each subcluster. Differentially expressed genes in each cluster were analyzed using Metascape (https://metascape.org/), and GO terms related to cartilage development were selectively examined with an enrichment score cutoff of –Log (p-value) ≥ 5. In the GO term panel, A represents ECM and tissue organization, B: cytoskeleton and cell structure, C: cell migration and adhesion, D: signal transduction, E: chondrogenesis, F: muscle differentiation, and G: homeostasis. The significance of enrichment is represented by –log (p-value). **e)** Key cartilage-related genes, including *COL2A1*, *ACAN*, *MATN4*, *SOX9*, *COMP*, and *PTN*, show concentrated expression in cluster 0 and 1 on the UMAP. **f)** The number of signaling interactions between all clusters identified through CellChat analysis. **g)** Ligand-receptor-based signaling analysis across clusters. Cell-cell interaction communication patterns among clusters were interrogated using CellChat analysis, and the relative strength of all enriched signals (both outgoing (left panel) and incoming (right panel)) across all clusters was visualized in a heatmap. **h)** Gene network between primary ligands identified in MIUChon CL2—COLLAGENs, *FN1*, and *PTN*—and their corresponding receptors. Ligand–receptor interactions involving COLLAGEN, *FN1*, and *PTN*, as predicted by CellChat analysis, were evaluated using STRING (https://string-db.org/) with a high confidence score (≥ 0.7). These interactions were further analyzed using Cytoscape (www.cytoscape.org) based on the degree of node connectivity within the gene network. **i)** Functional annotation of the genes comprising the gene network. Functional annotation of the ligand genes—COLLAGENs, *FN1*, and *PTN*—and their corresponding receptors was performed using STRING in conjunction with the Reactome pathway database.

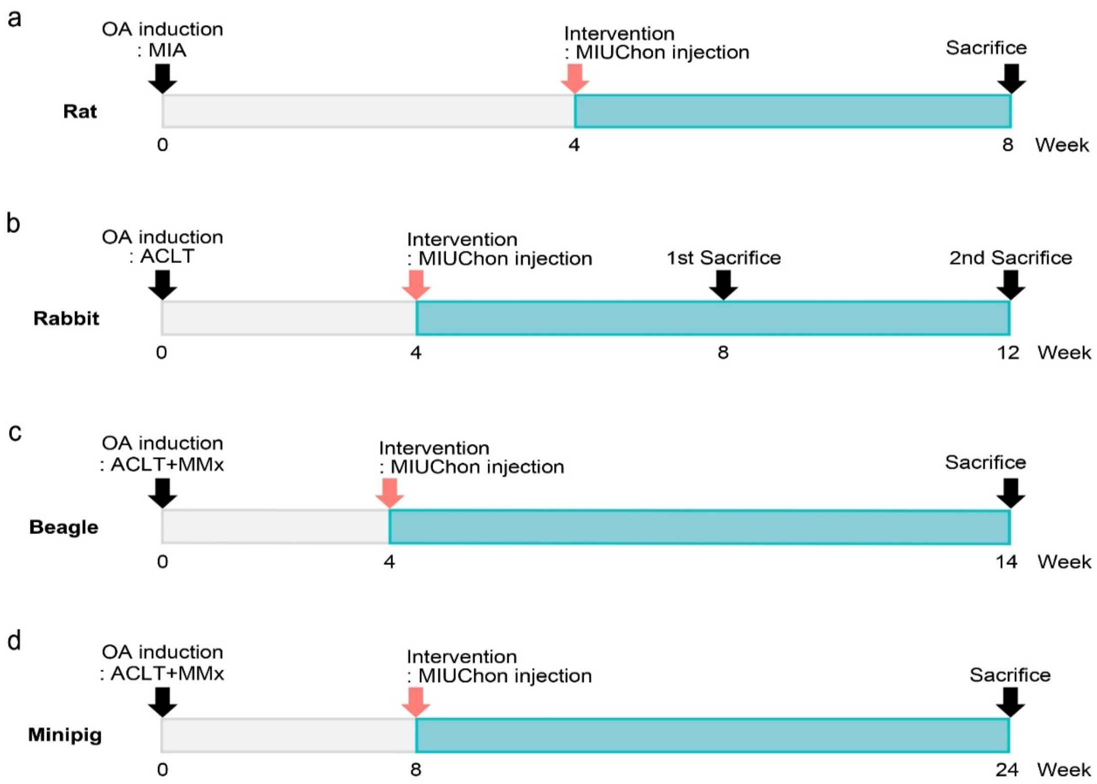

Supplementary Figure 8. Animal experiment scheme in **a**) rats, **b**) rabbits, **c**) beagles, and **d**) minipigs.

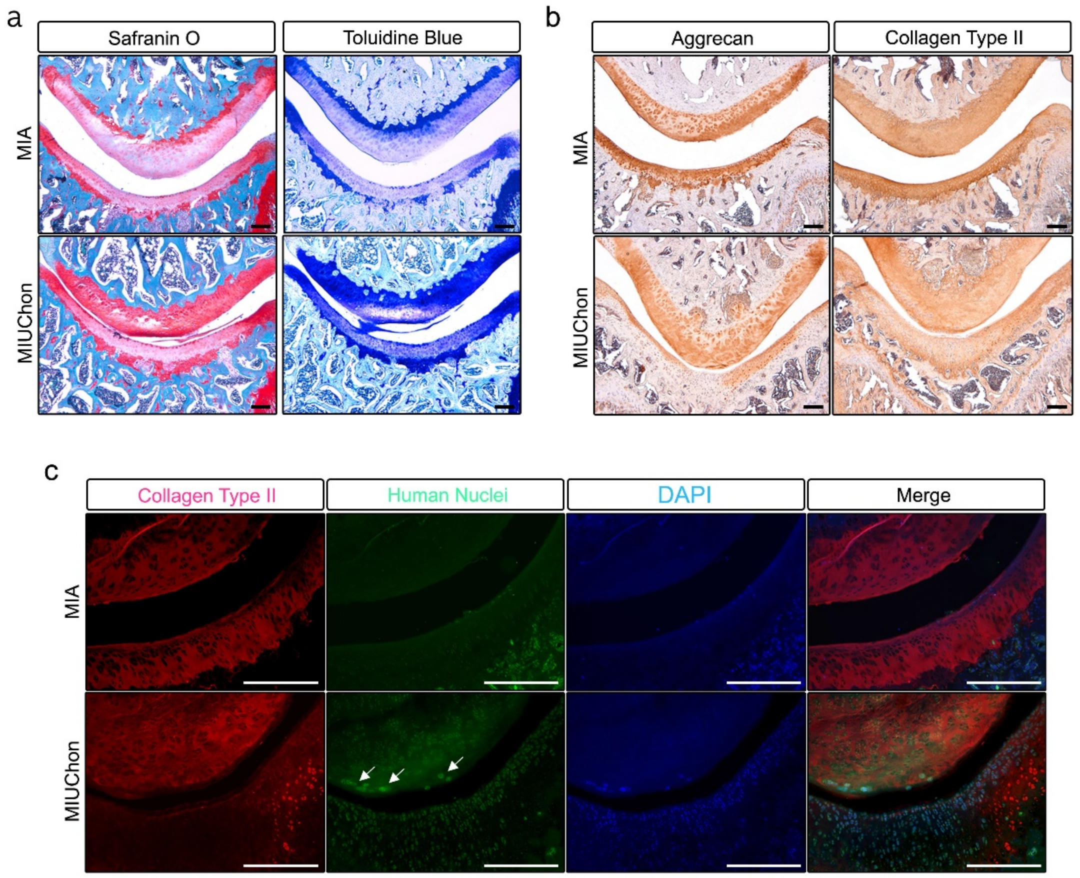

Supplementary Figure 9. Histological analysis of MIUChon-injected rat articular cartilage. Images of cartilage in the femur and tibia at 8 weeks post-injection. **a**) Preserved cartilage confirmed with safranin O and toluidine blue staining. **b**) Images showing staining against aggrecan and type II collagen. **c**) Immunofluorescence staining images confirming type II collagen and human nuclei expression. Scale bars indicate 200 μm.

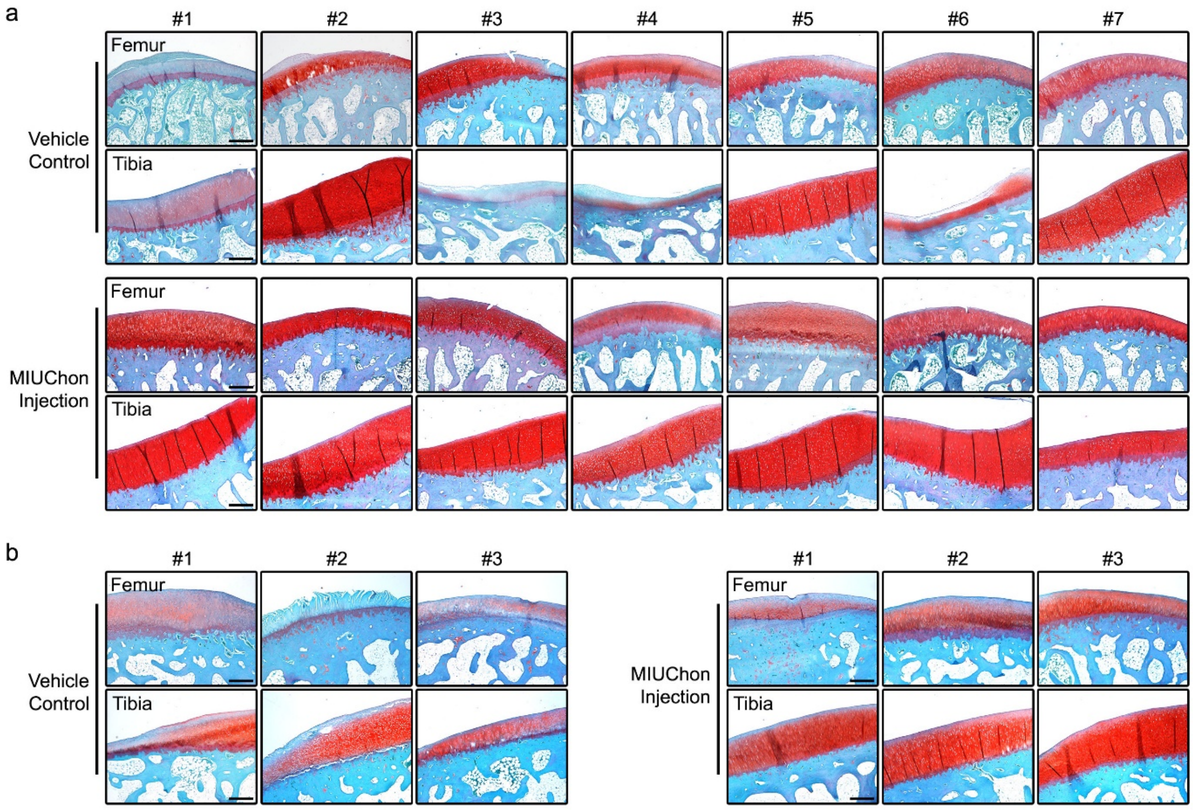

Supplementary Figure 10. Histological analysis of MIUChon-injected rabbit articular cartilage. Images of cartilage in the femur and tibia at **a**) 4 and **b**) 8 weeks post-injection. Scale bars indicate 200 μm.

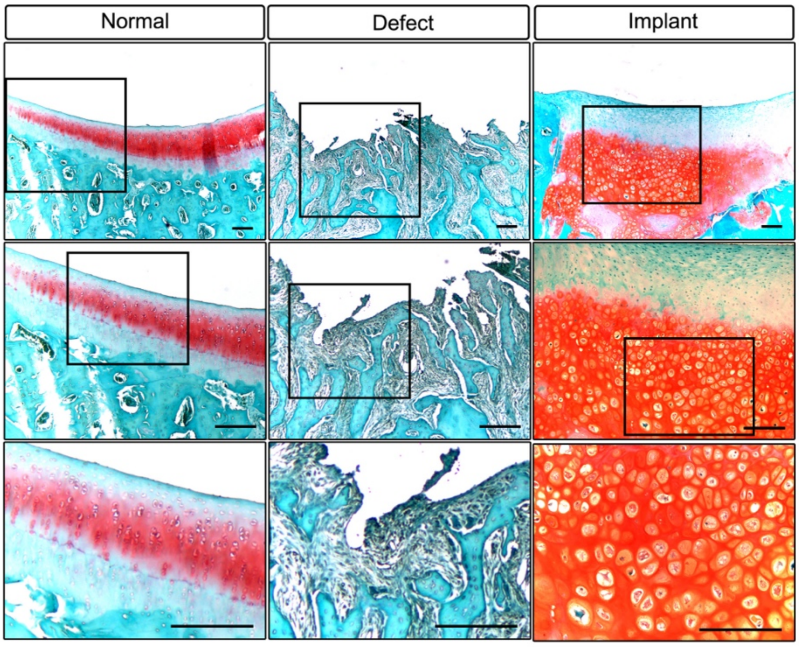

Supplementary Figure 11. Merge and regeneration test of MIUChon in the rabbit cartilage defect. Representative Safranin O-stained sections show Normal cartilage (left), an untreated Defect (middle), and an MIUChon-Implant site (right) harvested 1 week after implantation. In the Implant column, a Safranin O–positive MIUChon region is present within the defect; the faint layer overlying this region likely represents early provisional repair tissue (e.g., fibrous matrix/residual fibrin/host-derived provisional matrix) typical of the early healing window, and therefore the surface does not yet resemble mature hyaline cartilage. Scale bars indicate 200 μm.

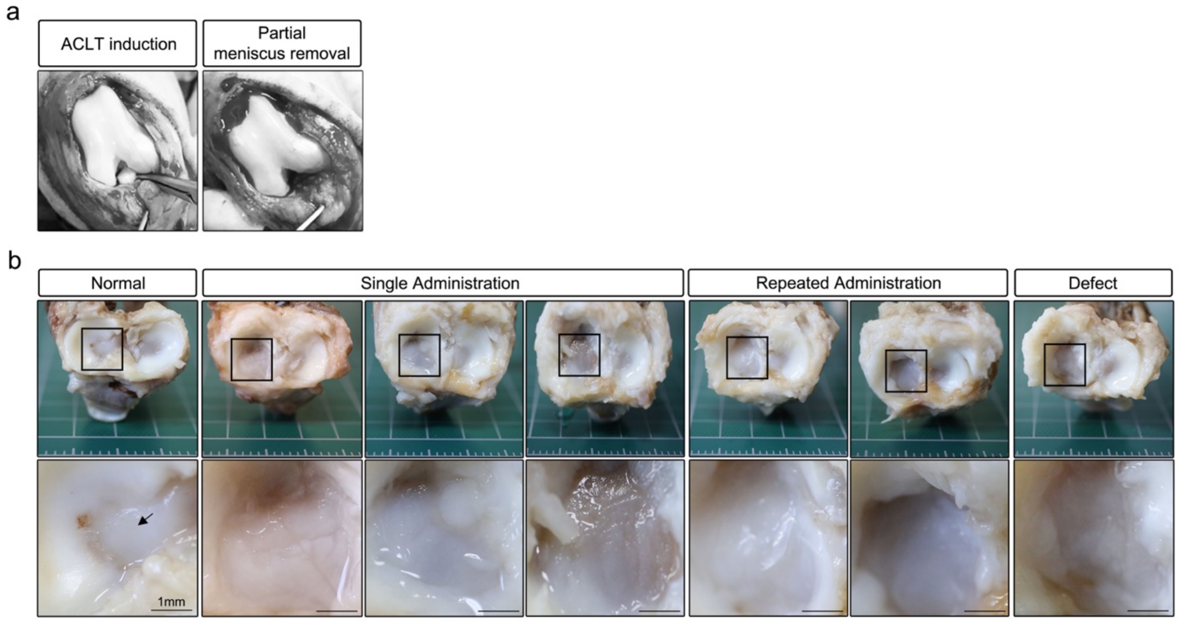

Supplementary Figure 12. The tibia of beagle OA models. The images show the removed left meniscus.

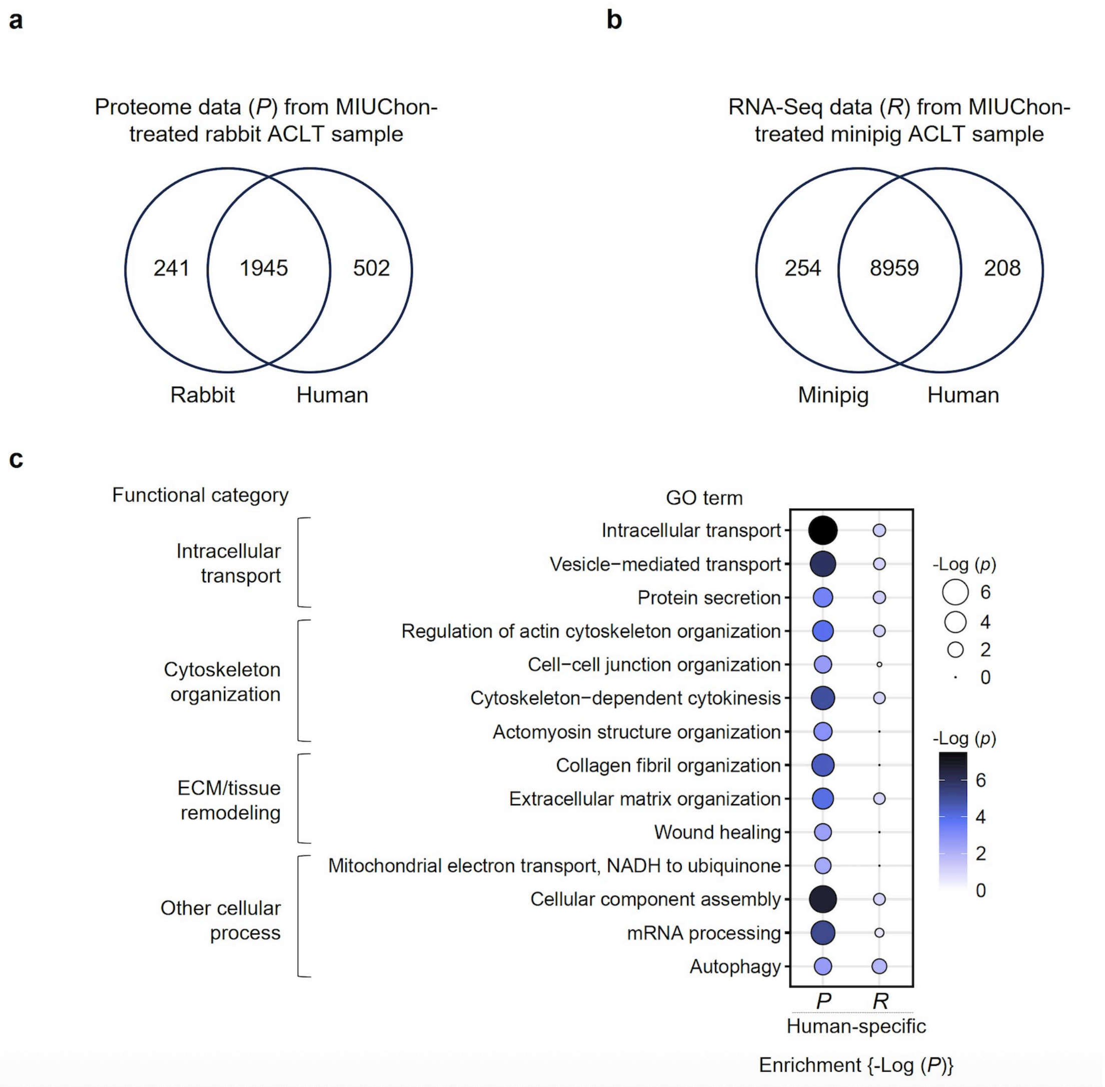

Supplementary Figure 13. Human-specific transcripts and proteins identified in MIUChon-treated ACLT animal models (rabbit and minipig). a) Venn diagram of proteome data (P) from MIUChon-treated rabbit ACLT samples. Protein sequences identified by LC-MS/MS were aligned to both rabbit and human reference proteomes, and classified into rabbit-specific proteins (241), human-specific proteins (502), and shared proteins (1,945; including orthologous proteins detected in both species). **b)** Venn diagram of RNA-Seq data (R) from MIUChon-treated minipig ACLT samples. RNA-Seq reads were aligned separately to the minipig and human reference genomes, and expression levels were quantified. Species-specific transcripts were defined as genes with uniquely mapped reads and expression restricted to only one reference genome—either human (208 genes, detected only in MIUChon-treated groups) or minipig (254 genes). A total of 8,959 transcripts were commonly mapped to both species. **c)** Gene Ontology (GO) enrichment analysis of human-specific molecules identified from MIUChon-treated ACLT animal models (rabbit and minipig). Functional categories include intracellular transport, cytoskeleton organization, extracellular matrix (ECM)/tissue remodeling, and other cellular processes. Representative enriched GO terms are shown, with dot size corresponding to significance (–Log *p*) and color intensity indicating enrichment level.

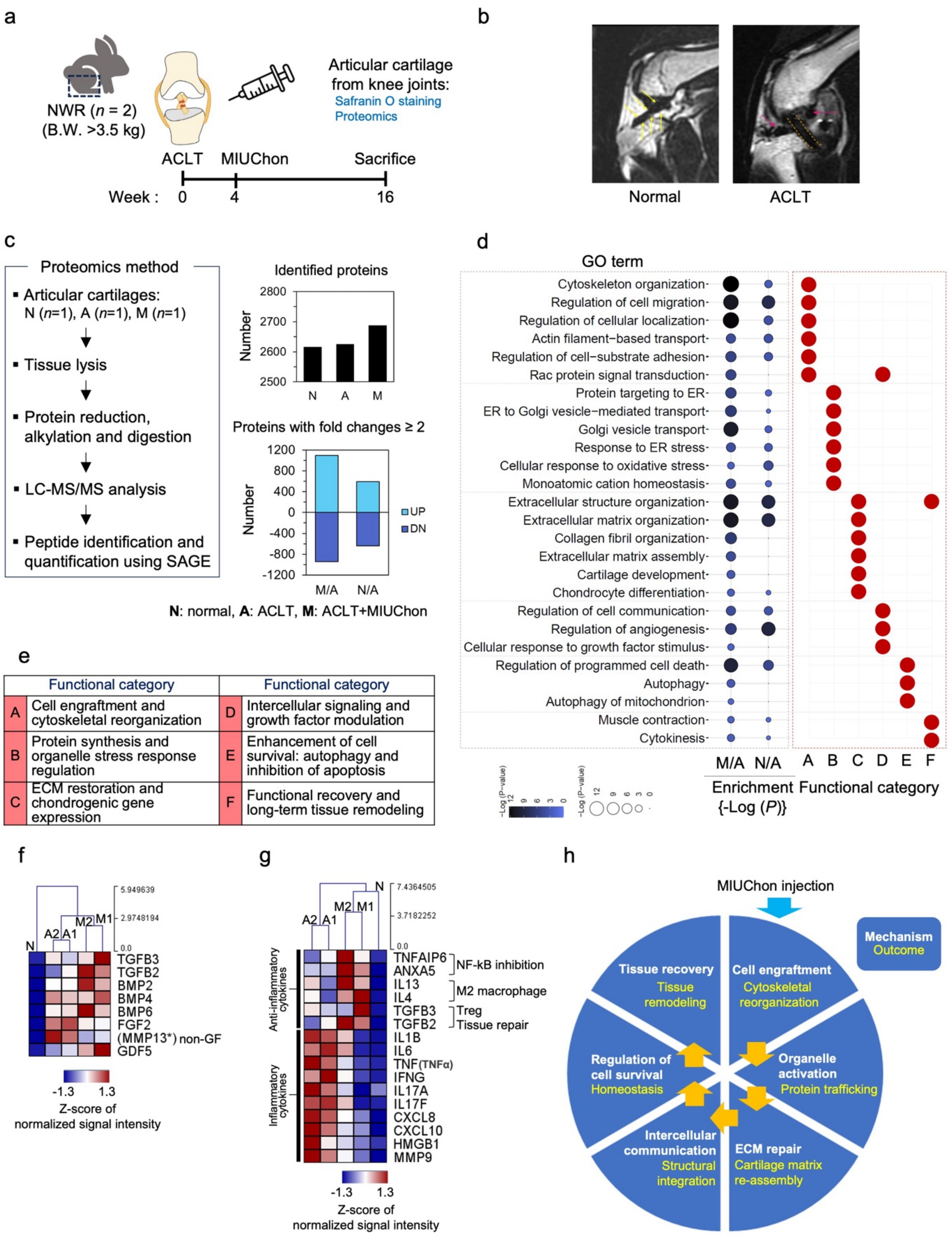

Supplementary Figure 14. Mechanistic insight into MIUChon action in a rabbit ACLT-induced osteoarthritis model based on proteomic profiling. a) Schematic of the experimental timeline. Osteoarthritis was induced in rabbit knee joints (n = 2) via ACLT (anterior cruciate ligament transection), followed by intra-articular injection of MIUChon at 4 weeks. Animals were sacrificed at 16 weeks, and articular cartilage was harvested for histological and proteomic analysis. **b)** MRI images confirming successful induction of the ACLT model compared to normal cartilage. **c)** Overview of the proteomics workflow. Cartilage samples from the three

groups (N, A, M) were processed for protein extraction, alkylation, digestion, and LC-MS/MS-based proteome profiling. The number of identified proteins in each group is shown. Proteins with ≥2-fold changes between M/A and N/A were quantified, and the upregulated proteins were used for downstream gene ontology (GO) enrichment analysis. **d)** GO enrichment analysis of proteins upregulated in the MIUChon-treated and normal groups relative to the ACLT group. The analysis was performed using DAVID with an EASE cutoff of < 0.01. Enriched terms were associated with cartilage development, ECM organization, and tissue regeneration. **e)** Functionally enriched GO terms were categorized into six major mechanistic categories: (*i*) cell engraftment and cytoskeletal reorganization, (*ii*) protein synthesis and organelle stress response regulation, (*iii*) ECM restoration and chondrogenic gene expression, (*iv*) intercellular signaling and growth factor modulation, (*v*) enhancement of cell survival, and (*vi*) functional recovery and long-term tissue remodeling. **f)** Heatmap showing the protein expression patterns of cartilage-related growth factors in synovial fluid from normal (N), ACLT (A1 and A2), and MIUChon-treated (M1 and M2) rabbit knee joints, as assessed by the RayBiotech Rabbit L1 Array. **g)** Heatmap showing the protein expression of inflammation-associated cytokines in synovial fluid from normal (N), ACLT (A1 and A2), and MIUChon-treated (M1 and M2) joints. **h)** Schematic model summarizing the proposed mechanism of action (MoA) of MIUChon in the ACLT model. MIUChon promotes cell engraftment, cytoskeletal reorganization, ECM repair, intercellular communication, and tissue recovery.

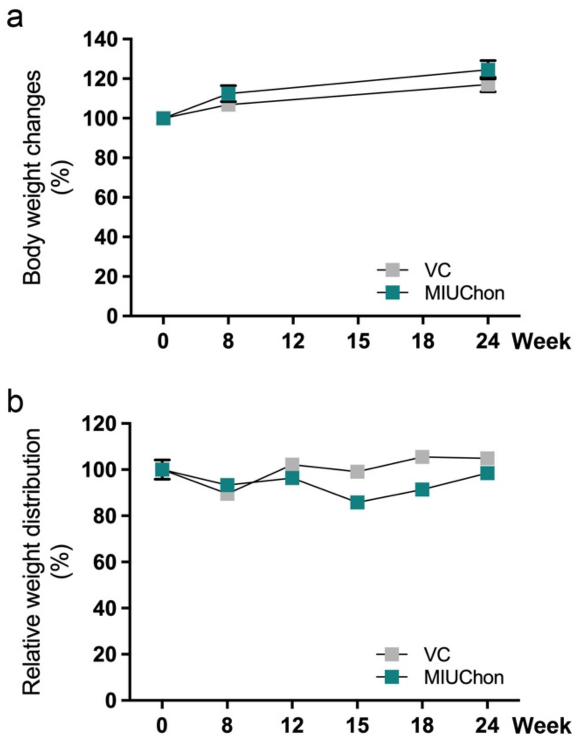

Supplementary Figure 15. Weight changes in MIUChon-injected minipigs.

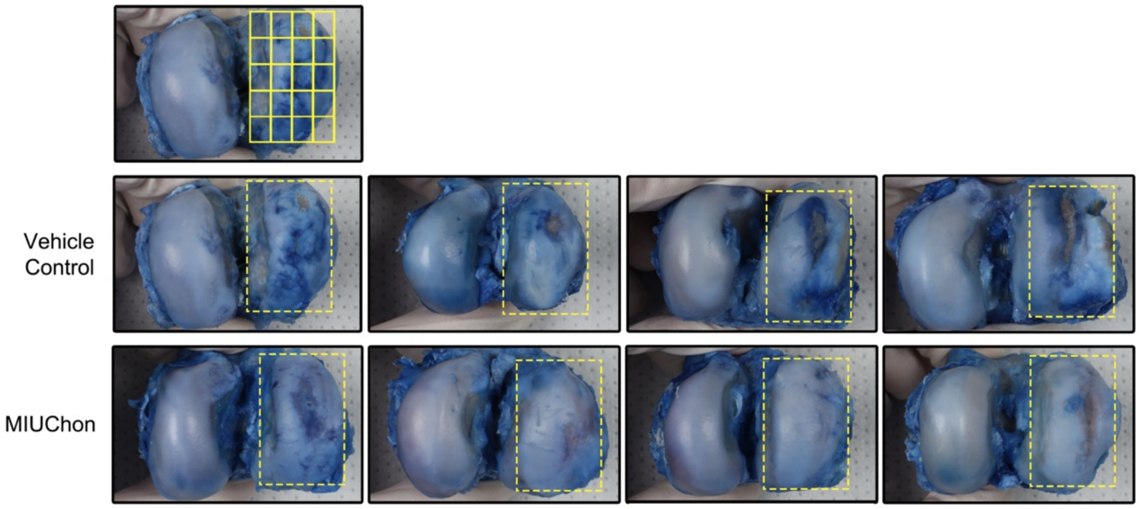

Supplementary Figure 16. The analyzed region in the minipig joint cartilage.

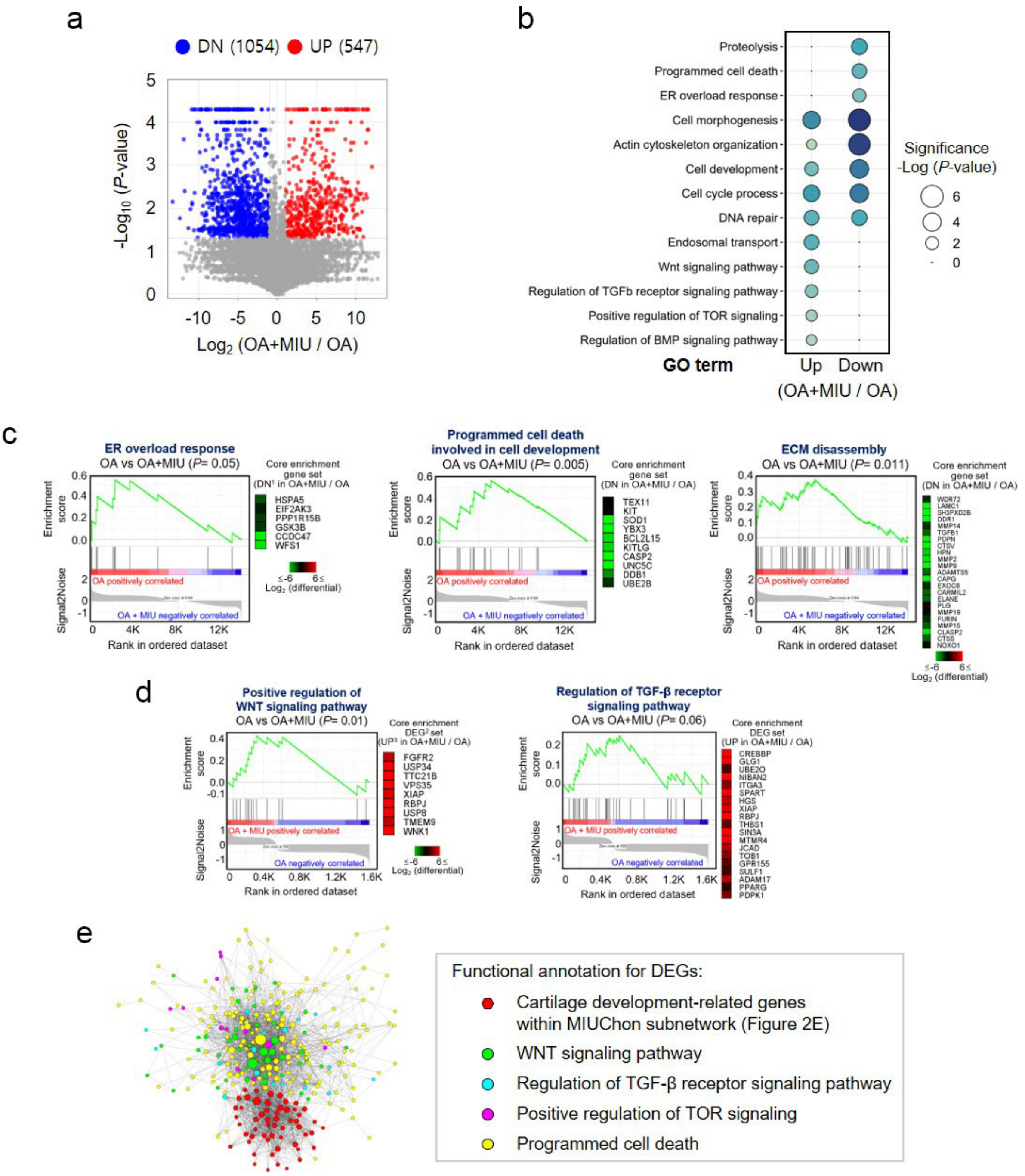

Supplementary Figure 17. Transcriptome analysis of MIUChon-treated OA samples in minipigs. **a**) Volcano plot of differentially expressed genes (DEGs) between OA-induced (n=5) and MIUChon-treated (n=5) groups. RNA-Seq was performed on paraffin block samples from both OA-induced and MIUChon-treated groups, followed by DE analysis between the two groups with with the cutoff set at P < 0.05 and a ≥ |1.5|-fold change. DN, down-regulated genes; UP, up-regulated genes; NS, non-significantly changed genes. **b**) GO enrichment analysis for DEGs. DEGs were annotated using DAVID, and significant GO terms were selected with a cutoff of EASE score P < 0.01. **c**) Enrichment plot for 'ER overload response' (P= 0.05), 'programmed cell death involved in cell development' (P= 0.005), and 'ECM disassembly' (P= 0.011). The top portion of the plot shows the running enrichment score (ES) for the gene set as the analysis continues down the ranked list (middle portion of the plot). GSEA was applied with a background dataset consisting of 14100 expressed genes. In core enrichment gene set,

down-regulated genes in MIUChon-treated group were visualized with expression heatmap. **d**) Enrichment plot for positive regulation of 'WNT signaling pathway' (P= 0.01) and 'regulation of TGF-β receptor signaling pathway' (P= 0.06). GSEA was applied with a background dataset consisting of 1,601 DEGs. In core enrichment gene set, up-regulated genes in MIUChon-treated group were visualized with expression heatmap. **e**) STRING network analysis of DEGs related to WNT signaling pathway, TGF-β receptor signaling pathway, TOR signaling, Programmed cell death coupled with cartilage development-related genes within MIUChon subnetwork (Figure 2e). Interaction of genes related to those functional signaling pathways/categories was evaluated using the STRING database (https://string-db.org/), with a high confidence score (≥0.7). This interaction was further analyzed using Cytoscape (www.cytoscape.org) based on the degree of connectivity of the nodes.

Supplementary Table 1. Characterization of the established iPSC bank.

LAL, Limulus amebocyte lysate; CPE, Cytopathic effect; HAD, Hemadsorption; HA, Hemagglutination; F-PERT, Fluorescent-PCR-based reverse transcription

Test item	Test method	Criteria
Morphology	Microscopic observation	Colony-forming cells with a large nucleus
Proliferation	MTT assay	72-hr analysis
	Gene expression	
	qPCR	Positive expression of LIN28, NANOG, OCT4, and SOX2
Undifferentiation markers	Protein expression	
	Immunocytochemistry	Positive expression of LIN28, NANOG, SSEA4, and TRA-1-60
	Alkaline phosphatase staining	Positive purple staining in colonies
Pluripotency	3-germ layer differentiation	Positive expression of PAX6, T, and SOX17 in differentiated cells
Purit (Residual exogenous TFs)	qPCR	Negative expression of Sendai virus, OCT4, SOX2, KLF4, and c-Myc
Genomic stability	CytoscanHD	No abnormalities detected
	Chromosome analysis	No abnormalities detected
	STR	No abnormalities detected
	CNV	No abnormalities detected
Sterility	Membrane filter	Negative
Mycoplasma	PCR	Negative
Endotoxin	LAL turbidimetry test	Negative
Adventitious viruses	*In vitro* assay	Negative for CPE, HAD, and HA
	In vivo assay	Negative after inoculation of adult mice, suckling mice, and embryonated chicken eggs
Other viral safety		
Bovine virus test		Negative
Porcine virus test		Negative
Retro virus test	TEM	Negative
	F-PERT	Negative
Human virus test		Negative

Supplementary Table 2. Characterization of the final MIUChon product.

Test item	Test method	Criteria
Morphology	Microscopic observation	150–200 μm white chondrogenic spheroids
Cell number	Total cell count	$5.64 \times 10^5 \pm 20\%$
Viability	Cell count with Trypan blue stain	≥70%
Identification test	Flow cytometry	≥70% expression of CD44 and COL2
Purity test	qPCR for LIN28, OCT4, NANOG, and SOX2	Ct value ≥ 35 ± 5
Potency test	Colorimetric assay for sGAG	≥3 μg per 200 MIUChon
Impurity test	ELISA for BSA	Negative
Sterility	Membrane filter	Negative

BSA, Bovine serum albumin

Supplementary Table 3. Parameters of the scRNA-seq data. Sequencing data parameters were obtained using 10X Cell Ranger software. Both analyzed samples contained 2,000–4,000 cells per sample.

			MIUChon			
Parameter	MCB	OGC	Day 3	Day 7	Day 13	Day 14
Number of Cells	10,960	3,195	24,394	22,682	5,679	7,969
Number of Reads	192,469,287	150,619,239	632,327,236	460,483,076	504,230,757	450,263,138
Valid Barcodes (%)	97.9	96.5	97.6	97.6	96.9	97.7
Mean Reads/Cell	17,561	47,142	25,921	22,682	88,789	56,502
Median Genes/Cell	1,966	3,781	2,888	2,250	6,618	5,787
Median UMI Counts/Cell	3,840	11,312	8,372	5,626	37,959	27,803

Supplementary Table 4. Diagonal weight bearing distribution (DWBD)(%).

	Vehicle control Group				MIUChon Group			
	**1**	**2**	**3**	**4**	**1**	**2**	**3**	**4**
**0-wk**	37.84907	37.07896	36.65726	37.34827	36.93586	36.51515	29.09091	37.89007
**8-wk**	32.6049	39.02439	28.91607	27.41703	34.94128	29.56562	27.88462	27.22549
**12-wk**	32.72496	36.51399	27.3762	27.37624	39.35897	33.67161	37.55734	34.52808
**15-wk**	28.16742	33.68182	32.64225	33.48083	35.37945	34.61712	38.48677	31.23381
**18-wk**	31.0124	43.50421	31.41593	34.16347	39.28479	38.00479	40.75846	36.98887
**24-wk**	31.06604	40.46296	33.36769	35.82219	37.32729	36.89817	37.80561	37.40014

Supplementary Table 5. Individual ∆ of diagonal weight bearing distribution (%, ∆DWBD).

	**Vehicle control Group**					**MIUChon Group**				
	**1**	**2**	**3**	**4**	**Average**	**1**	**2**	**3**	**4**	**Average**
**12-wk**	0.12	-2.51	-1.54	-0.04	-0.99	4.42	4.11	9.67	7.3	6.37
**15-wk**	-4.44	-5.34	3.73	6.06	0	0.44	5.05	10.6	4.01	5.03
**18-wk**	-1.59	4.48	2.5	6.75	3.03	4.34	8.44	12.87	9.76	8.85
**24-wk**	-1.54	1.44	4.45	8.41	3.19	2.39	7.33	9.92	10.17	7.45
**Average of 4 point**					1.3075					6.925

Supplementary Table 6. GO enrichment results for Supplementary Figure 14d.

Functional category (as manual)	GO ID	GO term	M (ACLT+MIUChon) / A (ACLT)					N (Normal) / A (ACLT)				
			P-value	-Log (*P*-value)	Hits (Genes)	No. of hit	Fold Enrichment	*P*-value	-Log (*P*-value)	Hits (Genes)	No. of hit	Fold Enrichment
1. Cell engraftment and cytoskeletal reorganization	GO:0007010	Cytoskeleton organization	5.30E-13	12.276	NCKAP1, ARPC5L, GOLGA2, STMN1, DAG1, SOX9, PLS1, AGFG1, TPM4, ACTN1, SWAP70, TPM2, TPM1, SPICE1, KRT5, ACTN4, EML1, EML2, CLIP1, MAP1B, MAP1A, TAGLN2, TLN1, PFN2, MACF1, SPTBN4, SHC1, FLII, CUL3, IQGAP1, CORO1B, CAMSAP2, CORO1C, KIF3B, KIF3A, CALD1, EPB41L2, EPB41L3, PACSIN2, MYH11, MAP4, SPTBN1, GFAP, SMTN, SON, ARPC2, TRPV4, TBCE, NF1, GRB2, ITGB1, NUMA1, TGFB1I1, BRK1, FHL3, WASL, BRCA1, SYNE3, MED18, CAPZB, RAC1, SPTAN1, CGNL1, NCK1, CAP1, ACTR3, SUN2, ACTR2, MYBPC1, NUDC, ANXA1, RHOG, NAV1, ENAH, DDB1, ACTA2, PALLD, CDC42EP2, HCLS1, MYH9, MAPRE3, ANG, CASQ1, LCP1, ARHGEF2, MAPRE1, MYH7, ARF6, KANK1, ROCK1, DCTN1, ABRAXAS2, AIF1, LIMA1, LMAN2, FLNB, FLNC, DCAF13, RANBP1, MARCKSL1, GSN, SPAG5, CSNK1A1, RAB11A, MYO1D, MYO1E, MYO1B, MYO1C, CENPH, CAPZA2, ZYX, CTNNB1, KRAS, EPPK1, MYO1F	115	2.023	0.001497055	2.825	MTPN, SPTBN4, KANK1, NCKAP1, GMFB, TMOD2, RDX, LMOD1, APOA1, ADD3, SORBS3, LIMA1, CDC42EP2, GRB2, SYNPO, CGNL1, SPTBN1	17	2.485
	GO:0030334	Regulation of cell migration	1.18E-11	10.929	ITGB1, PLVAP, ECM1, TNFAIP6, TRADD, PDCD6, MIA3, PTPN23, WASL, CRKL, ICAM1, SMPD3, MECP2, LGALS3, GLIPR2, AKT2, AKT3, DAG1, CYP1B1, CPNE3, EMILIN1, SOX9, TIMP1, LBP, RAC1, NCK1, MAP2K3, PDGFRB, SUN2, ANXA1, SWAP70, TPM1, RHOG, ACTN4, MIF, RHOB, SFRP2, MMRN2, PECAM1, MYADM, NUMB, CDH13, ITGA6, ARF6, PFN2, LRRC15, KANK1, GRN, ROCK1, CD99L2, PTN, PLA2G7, AIF1, RTN4, CORO1C, GNA13, SDCBP, ATP5F1B, GPNMB, HMOX1, STX4, CCL19, RARRES2, RDX, STAT3, LAMB1, PODN, RAB11A, COL1A1, DAB2, MYO1C, PTPRC, AGO2, NF1	74	2.370	5.98E-09	8.223	KANK1, APP, HDAC5, ECM1, PLVAP, SEMA3C, SEMA3B, HSPB1, MIA3, THY1, PTN, AIF1, CORO1C, MECP2, GNA13, SMPD3, SDCBP, DDRGK1, AKT2, BSG, DAG1, PLCG2, CYP1B1, STX4, TIMP1, GLUL, SUN2, RARRES2, CAV1, RDX, TPM1, ADIPOQ, RRAS2, LAMB1, ACTN4, LYVE1, RHOB, DAB2, SFRP2, AGO2, MMRN2, NF1, CDH13, ADA, ENG	45	2.680
	GO:0060341	Regulation of cellular localization	6.02E-13	12.221	ITGB1, GSK3A, NUMA1, TNFAIP6, CTNND1, CLTC, ARHGAP1, PTPN23, WASL, IPO7, LGALS3, SCP2, SUMO1, TRIM28, AKT2, DAG1, ARFIP1, EMD, AP2M1, PLS1, FGB, SWAP70, STX7, RHOG, P3H1, RANGAP1, VAMP8, PTP4A3, MAP1B, PECAM1, NUMB, HCLS1, TAX1BP3, ANG, LCP1, ISCU, ARF6, YAP1, LRRC15, KHDRBS1, CAMK2D, TMED10, DHX9, GNAI3, MACROH2A1, GNAI1, RTN4, TM9SF4, SNX3, PGRMC1, SCFD1, CNPY4, BAG3, EPB41L2, TPR, STOM, STX4, SPTBN1, BTF3, GSN, SEC24A, SPAG5, RDX, RAB11A, DAB2, UFM1, MYO1C, NF1, BAX, PTPN9, CTNNB1, GOPC, ANP32B, CAMK1, PFKM, EIF4G1	76	2.484	1.37E-04	3.863	CTNND1, IPO7, MACROH2A1, TM9SF4, RAP1A, SUMO1, TRIM28, LAMP1, DDRGK1, BAG3, ALOX5, AKT2, DAG1, CHP1, STX4, CD36, GLUL, SPTBN1, PPP1R12A, SPAG5, B3GAT3, CAV1, RDX, ADIPOQ, P3H1, RANGAP1, LZTFL1, PML, NSFL1C, DAB2, NF1, CAMK1, PFKM, ISCU	34	2.058
	GO:0099515	Actin filament-based transport	4.94E-05	4.306	MYO1D, MYO1E, SUN2, MYO1B, MYO1C, ACTN4, MYO1F	7	9.527	1.67E-04	3.777	MTPN, SPTBN4, KANK1, NCKAP1, GMFB, CAV1, TMOD2, RDX, LMOD1, APOA1, ABRACL, ADD3, SORBS3, LIMA1, SUMO1, CDC42EP2, GRB2, SYNPO, CGNL1, SPTBN1	20	2.713
	GO:0010810	Regulation of cell-substrate adhesion	4.10E-06	5.387	VIT, MACF1, KANK1, COL16A1, ROCK1, RHOG, CASK, ACTN4, TRIOBP, CORO1C, CRKL, COL1A1, ARPC2, NF1, MYADM, CDH13, COL8A1, EMILIN1, MELTF, RAC1, UTRN, LIMS1	22	3.237	3.10E-04	3.509	VIT, MACF1, KANK1, COL16A1, APOA1, ACTN4, THY1, TRIOBP, CORO1C, NF1, CDH13, MELTF, CD36	13	3.557
	GO:0016601	Rac protein signal transduction	6.63E-06	5.179	CYFIP1, NCKAP1, NF1, RHOG, BRK1, CDH13, KRAS, AIF1	8	10.058	-	-	-	-	-
2. Protein synthesis and organelle stress response regulation	GO:0045047	Protein targeting to ER	3.36E-06	5.473	BAG6, SEC61A1, SPCS3, SPCS2, SRP72, SSR3, SRPRA, CHMP4A, SRP68, SEC61B, SRP9	11	6.654	0.011504925	1.939	BAG6, SEC61A1, SPCS2, SRP72, SRP68	5	5.625
	GO:0006888	ER to Golgi vesicle-mediated transport	8.08E-07	6.092	COPB2, SEC23A, COPA, TMED10, SEC24A, CUL3, COPB1, PDCD6, MIA3, YIF1B, LMAN1, SCFD1, SEC23IP, TFG, VAPB, LMAN2, HYOU1, COPG1, SEC24D, TMED7, TMED5, TMED4, SEC31A	23	3.448	0.151895971	0.818	SEC13, VAPB, LMAN2, MIA3, SEC23B, CNIH4, YIF1B	7	1.944
	GO:0048193	Golgi vesicle transport	4.90E-11	10.310	COPB2, COPA, SEC23A, TMED10, CUL3, COPB1, PDCD6, GOSR2, MIA3, SNX3, LMAN1, SNX1, GOLGA4, SCFD1, TFG, AP1G1, LMAN2, ANKFY1, RAB6A, TMED7, CCDC93, EPS15, TMED5, SPTBN1, RAB8B, SEC31A, TMED4, SEC24A, COPZ2, YIF1B, RUFY1, MYO1B, SEC23IP, EHD3, VAPB, RAB34, RAB13, HYOU1, COPG1, SEC24D, EXOC2, EXOC1, VPS35L, VAMP3	44	3.152	0.00377824	2.423	SEC13, COG5, COPZ2, MIA3, YIF1B, ARFGAP2, MYO1B, LAMP1, VAPB, RAB34, LMAN2, SEC23B, EPS15, CNIH4, SPTBN1, VAMP3, EXOC1	17	2.265
	GO:0034976	Response to ER stress	7.12E-05	4.148	DDX3X, ATP2A3, EIF2AK2, ERLIN2, PDIA6, DNAJC3, BAG6, UFM1, RNF103, NPLOC4, VAPB, DNAJC10, CANX, UFC1, BAX, UFD1, HYOU1, P4HB, CALR, UBXN4, UGGT2, NCK1, EIF4G1	23	2.617	8.07E-04	3.093	BAG6, OPA1, DDRGK1, CAV1, ALOX5, AKT2, CLU, UBQLN2	8	5.225
	GO:0034599	Cellular response to oxidative stress	0.004595916	2.338	ANXA1, TPM1, PYCR1, PYCR2, AIF1, OXR1, RHOB, SMPD3, NAGLU, PRKRA, ARL6IP5, CYP1B1, ADPRS, STX4, SLC25A24	15	2.361	4.09E-05	4.388	G6PD, SRXN1, TPM1, APOA4, AIF1, RHOB, MAPK13, SMPD3, NAGLU, CYP1B1, ADPRS, STX4, CD36, SLC25A24	14	4.081
	GO:0055080	Monoatomic cation homeostasis	3.56E-05	4.448	GRN, STOML2, STEAP4, HEXB, ATP2A3, SLC4A1, ITPR3, ATP1A1, TM9SF4, BOLA2B, NT5E, ATP5F1B, SGCD, NAGLU, ANKH, FTH1, LAMP2, TRIM3, NPTN, ANXA6, HMOX1, SLC39A7, SLC12A7, ATP6V0A1, SLC12A4, SCARA5, IMMT, ATP1B3, ATP2B1, ATP1B1, CP, TF, VAPB, TRPV4, PPT1, ALPL, BAX, ATP6V0D1, ISCU	39	2.065	0.015	1.837	APP, CAV2, CAV1, ITPR3, ATP2B1, TM9SF4, PML, BOLA2B, TF, SGCD, NAGLU, LAMP1, VAPB, LAMP2, PPT1, CHP1, SLC12A7, ISCU, TGM2	19	1.864
3. ECM restoration and chondrogenic gene expression	GO:0043062	Extracellular structure organization	1.50E-11	10.824	ITGB1, VIT, COL16A1, COL11A2, LOXL1, FBLN5, RIC8A, SMPD3, ADAMTS2, PRDX4, ADAMTSL4, ADAMTSL3, DAG1, SERPINH1, CYP1B1, MYH11, EMILIN1, SOX9, POSTN, CRTAP, LAMB1, GFAP, COL1A1, MYO1E, MMP14, SFRP2, COL1A2, COL2A1, COL5A3, SMOC1, COL5A2, PXDN, NF1, COL9A1, COL21A1, LCP1, COL9A2, MATN3	38	3.666	2.23E-09	8.652	VIT, APP, COL16A1, FBLN5, RIC8A, SMPD3, ADAMTS5, ADAMTSL4, ADAMTSL3, DAG1, CYP1B1, CAV2, CAV1, LAMB1, GFAP, MYO1E, MMP14, SFRP2, COL4A1, SMOC1, COL5A2, PXDN, NF1, COL9A2, FMOD	25	4.468
	GO:0030198	Extracellular matrix organization	1.71E-11	10.766	ITGB1, VIT, COL16A1, COL11A2, LOXL1, FBLN5, RIC8A, SMPD3, ADAMTS2, PRDX4, ADAMTSL4, ADAMTSL3, DAG1, SERPINH1, CYP1B1, MYH11, EMILIN1, SOX9, POSTN, CRTAP, LAMB1, GFAP, COL1A1, MYO1E, MMP14, SFRP2, COL1A2, COL2A1, COL5A3, SMOC1, COL5A2, PXDN, NF1, COL9A1, COL21A1, LCP1, COL9A2, MATN3	38	3.645	2.27E-09	8.644	VIT, APP, COL16A1, FBLN5, RIC8A, SMPD3, ADAMTS5, ADAMTSL4, ADAMTSL3, DAG1, CYP1B1, CAV2, CAV1, LAMB1, GFAP, MYO1E, MMP14, SFRP2, COL4A1, SMOC1, COL5A2, PXDN, NF1, COL9A2, FMOD	25	4.460
	GO:0030199	Collagen fibril organization	4.95E-07	6.305	CRTAP, COL11A2, LOXL1, COL1A1, ADAMTS2, SFRP2, COL1A2, COL2A1, COL5A2, PXDN, NF1, SERPINH1, CYP1B1	13	6.395	-	-	-	-	-
	GO:0085029	Extracellular matrix assembly	3.89E-05	4.410	SMPD3, COL1A2, PXDN, EMILIN1, LAMB1, MYH11, SOX9, FBLN5	8	7.918	-	-	-	-	-
	GO:0051216	Cartilage development	5.96E-04	3.225	ECM1, COL11A2, THBS3, COL1A1, SMPD3, SFRP2, COL2A1, NFIB, MUSTN1, TRPV4, ANXA6, CTNNB1, SOX9, CNMD, CD44, MATN3	16	2.796	-	-	-	-	-
	GO:0002062	Chondrocyte differentiation	0.003317527	2.479	SMPD3, ECM1, SFRP2, COL2A1, MUSTN1, NFIB, COL11A2, ANXA6, CTNNB1, SOX9	10	3.263	0.089443522	1.048	SMPD3, ECM1, SFRP2, MUSTN1, DDRGK1, OGN, CNMD	7	2.266
4. Intercellular signaling and growth factor modulation	GO:0010646	Regulation of cell communication	4.81E-05	4.318	ACAA2, PDCD6, ITSN1, CTNND1, PEBP1, PHB2, CRKL, ICAM1, TIAL1, RPS15, LGALS3, RUVBL2, AKT2, AKT3, CPNE1, ARL6IP5, DAG1, CYP1B1, ENPP1, PIP4K2B, SOX9, FBXO7, RPS12, MAP2K3, PDGFRB, EPHA7, CSNK2A1, CASK, ANK2, ACTN4, MIF, SFRP2, FRMD6, RRAGC, TBL1XR1, PSME3, NCL, WDFY1, STAMBP, KCTD12, S100A8, PFN2, DDX5, SHC1, DDX1, CUL3, ITPR3, IQGAP1, AK7, GLG1, RTN4, STK3, SNX3, RAP1B, SDCBP, NCSTN, KIF3B, TSPAN6, TPR, HMOX1, SPTBN1, FIS1, PYCR1, EIF2AK2, PTK2, GFAP, COL1A1, DAB2, GCLC, NPLOC4, PTPRC, RHEB, NF1, PPT1, GRB2, HYOU1, RAB5A, ITGB1, GSK3A, ECM1, DDX3X, TNFAIP6, NUMA1, TRADD, FHL2, ADAR, PTPN23, BRCA1, YBX3, MED18, SMPD3, MECP2, GLIPR2, PEA15, TRIM3, NPTN, UFD1, EMILIN1, LBP, ERC1, TIMP1, EMD, CGNL1, TMED4, NCK1, FGB, ANXA1, MYOF, SAFB, ARFGAP1, EPN2, VAMP8, SFPQ, ELF1, MMP14, PTP4A3, COL2A1, MMRN2, MZB1, CHI3L1, HCLS1, CDH13, ITGA6, TAX1BP3, ARHGEF2, SLC25A4, CD44, RBMS3, ARF6, YAP1, CD63, KANK1, USP15, ROCK1, MVP, DDX21, PTN, GNAI2, PPP2CA,	159	1.353	3.32E-04	3.479	APP, SRPX, ECM1, ACAA2, CTNND1, APIP, HSPB1, NENF, LIMD1, YBX3, CLU, MED18, AKAP12, SMPD3, RPS15, MECP2, OPA1, RUVBL2, AKT2, PEA15, CPNE1, DAG1, NEFL, CYP1B1, FLOT2, CHP1, CD36, TIMP1, LMCD1, CGNL1, SEC13, EPHA7, TMOD2, ADIPOQ, APOA1, ACTN4, EPN2, DKK3, MMP14, ELF1, SFRP2, RRAGC, NCL, MMRN2, MZB1, CHI3L1, CDH13, VWA2, PFDN5, ENG, LTF, CD63, KANK1, USP15, DDX5, DDX1, ITPR3, PTN, NOL3, FXR1, SDCBP, NCSTN, BCLAF1, RAP1A, WNT11, DDRGK1, ALOX5, PLCG2, SNCG, STX4, SPTBN1, UBQLN2, CAV2, CAV1, EIF2AK2, SORBS3, PTK2, PML, GFAP, DAB2, RPS6KB1, FABP5, AXL, NF1, PPT1, PTPN6, GRB2, KRAS, GCLM, PFKM, ADA	91	1.435

					BCLAF1, RPS3, SNCG, SHOC2, STX4, CCL19, STAT1, CSNK1A1, STAT3, UFM1, PRKRA, AXL, PDCD4, BAX, CTNNB1, KRAS, LAMTOR2, PFKM, LAMTOR3, LIMS1							
	GO:0045765	Regulation of angiogenesis	1.38E-06	5.859	ITGB1, ECM1, ROCK1, PDCD6, RPN1, BRCA1, MECP2, STAB1, AKT3, CYP1B1, HMOX1, EMILIN1, CNMD, GTF2I, STAT1, STAT3, WARS1, RHOB, EPN2, TJP1, SARS1, SFRP2, AGO2, NF1, CHI3L1, CTNNB1, ITGA5	27	2.969	7.77E-10	9.109	ECM1, HSPB6, HSPB1, PML, WARS1, RHOB, EPN2, MECP2, SFRP2, ALOX5, TNMD, AGO2, STAB1, NF1, CHI3L1, CYP1B1, PTPN6, CD36, CNMD, CD34, GLUL, HTATIP2, TGM2, GTF2I	24	4.909
	GO:0071363	Cellular response to growth factor stimulus	0.009664808	2.015	DDX5, USP15, SHC1, IQGAP1, GLG1, CRKL, SMPD3, CPNE3, SOX9, EMD, MAP2K3, PDGFRB, ANXA1, STAT3, DAB2, MYO1C, SFRP2, COL1A2, COL2A1, NCL, PDCD4, ZYX, CTNNB1, GRB2, CD44	25	1.745	-	-	-	-	-
5. Enhancement of cell survival: autophagy and inhibition of apoptosis	GO:0043067	Regulation of programmed cell death	4.34E-10	9.362	ITGB1, USP36, RBM25, MTCH2, DDX3X, ACAA2, FHL2, ADAR, BRCA1, PEPD, PHB2, YBX3, ICAM1, MECP2, LGALS3, LGALS1, ADAMTSL4, FTH1, PEA15, ARL6IP5, CYP1B1, TMEM109, SOX9, SLC39A7, FBXO7, NCK1, FGB, CAST, EPHA7, ANXA1, SERPINB9, MIF, RHOB, DNAJC3, DDB1, SFPQ, SFRP2, COL2A1, PLSCR3, DAD1, PSME3, ALDH1A2, PTRH2, HCLS1, ADPRS, ITGA6, VDAC1, ANG, ITGA5, SLC25A4, CD44, S100A8, YAP1, KHDRBS1, GRN, USP15, ROCK1, DIABLO, SHC1, HSPD1, STK3, BAG6, BCLAF1, BAG3, RPS3, HMOX1, STX4, CCL19, ATG7, RBM10, HSPA9, FIS1, GSN, STAT1, PYCR1, EIF2AK2, OXR1, DAB2, GCLC, SON, PTPRC, MYBBP1A, FAP, PRKRA, AXL, CARM1, NF1, PDCD4, PPT1, BAX, CTNNB1, KRAS, HYOU1, ANP32B	94	1.968	1.22E-05	4.915	USP36, SRPX, ACAA2, HSPB6, APIP, HSPB1, PEPD, YBX3, CLU, MECP2, OPA1, FAM162A, ADAMTSL4, PEA15, NEFL, CYP1B1, TMEM109, HTATIP2, TGM2, EPHA7, TSC22D3, ADIPOQ, RPS3A, RHOB, DNAJC3, SFRP2, DAD1, ADPRS, VDAC1, USP15, DIABLO, NOL3, BAG6, BCLAF1, WNT11, BAG3, PLCG2, STX4, CAV1, EIF2AK2, PML, DAB2, RPS6KB1, AXL, NF1, PPT1, KRAS, CIAPIN1, GCLM, ADA	50	1.939
	GO:0006914	Autophagy	7.26E-04	3.139	WDR45B, VPS4B, HMGB1, PHB2, NAGLU, PPP1R8, LAMP2, TOM1, ANXA7, HMOX1, PIP4K2B, SNX7, FBXO7, ATG7, ATG3, HSPA8, FIS1, NIPSNAP3A, NIPSNAP2, VAMP8, UFM1, UFC1, CHMP2A, CHMP4A, S100A8	25	2.134	-	-	-	-	-
	GO:0000422	Autophagy of mitochondrion	0.029162059	1.535	FIS1, ATG3, NIPSNAP3A, WDR45B, SNX7, PHB2, FBXO7, ATG7, NIPSNAP2	9	2.466	-	-	-	-	-
6. Functional recovery and long-term tissue remodeling	GO:0006936	Muscle contraction	0.00795142	2.100	GSN, TPM4, MYL11, TPM2, TPM1, ANK2, ATP1B1, ACTA2, MYH3, SGCD, CALD1, TNNI2, MYH11, MYH7	14	2.301	0.095453856	1.020	FXR1, DES, NAGLU, CAV2, CAV1, TPM1, NF1, DAG1, KRAS	9	1.937
	GO:0000910	Cytokinesis	0.002559875	2.592	SON, ROCK1, VPS4B, DCTN3, CHMP2A, ANXA11, MYH9, CHMP4A, IQGAP1, STAMBP, SPTBN1, RHOB	12	2.926	0.180229339	0.744	CHMP2B, DCTN3, ANXA11, SPTBN1, RHOB	5	2.258
